# Supplementary material for: The pollen virome of wild plants and its association with variation in floral traits and land use
Source: Nat Commun. 2022 Jan 26;13:523. doi: 10.1038/s41467-022-28143-9 (PMC8791949; doi:10.1038/s41467-022-28143-9)
Supplement: Supplementary file 1 — Supplementary Information [file 41467_2022_28143_MOESM1_ESM.pdf]

Supplementary Methods

Pollen sample purity verification

We selected representative plant species that had either relatively low or relatively high estimates of pollen-associated virus richness for pollen sample purity verification. First, we assessed the level of physical contamination in pollen samples from three plant species using light microscopy. Second, we evaluated the potential for vegetative contamination based upon the expression level of pollen- and chloroplast-specific genes. We did this bioinformatically for two plant species using the RNAseq data generated herein as well as via real-time polymerase chain reactions (RT-PCR) using pollen and leaf RNA from one plant species.

1) Light microscopy

We added 600 µL of RNAse-free water (Zymo Research Corporation, Irvine, CA, USA) to extra pollen samples collected from *Packera aurea, Raphanus sativus,* and the *Solidago* species in 2018, which was collected in 2-mL tubes containing Lysing Matrix D (MP Biomedicals, Irvine, CA, USA). A similar tube with no pollen served as the control for this first analysis of pollen sample purity. After gently inverting the tubes several times, we viewed three ten-µL aliquots from the control and all three representative plant species with the aid of a Leica DM500 light microscope set at a magnification of 10X (Leica Microsystems, Buffalo Grove, IL, USA). A haphazardly chosen field-of-view of each control aliquot was photographed, and the most dense fields-of-view of all aliquots of each pollen sample were photographed with a Leica ICC50 W Camera (Module and Firmware versions 2016.1.0.6995 and 1.30.391676, respectively). We enumerated all pollen grains, pollen grain exine pieces, pollen intine or cytoplasm chunks, debris seen in the control (e.g., dust particles), and debris that was not seen in the control (i.e., true

biological contamination). Due to the low amount of contamination in the pollen samples, we show representative photographs of pollen from each plant species (Supplementary Methods Figure 1a). Overall, we found very few tissue fragments (*Packera aurea*: 2/153 pollen grains = 0.013% contamination; *Raphanus sativus*: 2/134 pollen grains = 0.015% contamination; *Solidago* sp.: 1/173 pollen grains = 0.006% contamination) that did not clearly originate from the pollen grains themselves (Supplementary Methods Figure 1a).

2) Pollen- and chloroplast-specific gene expression

We determined whether vegetative plant material, such as leaves or sepals, contaminated the pollen samples by evaluating the expression of pollen- and chloroplast-specific genes therein. Given that chloroplasts are generally only maternally inherited[1] and thus not incorporated in pollen grains, we reasoned that they would be good indicators of vegetative contamination. First, we assessed the potential for vegetative contamination bioinformatically using RNAseq data that we generated from *Fragaria chiloensis* and *Raphanus sativus* pollen. RNAseq data from *Arabidopsis thaliana* leaf tissue[2] was used as a standard of expression for the chloroplast-specific genes. Second, we further assessed the potential for vegetative contamination by performing an RT-PCR experiment on RNA from *Raphanus sativus*, a plant species from which we collected contemporary pollen and leaf samples and whose entire genome has been sequenced, to determine the expression of pollen- and chloroplast-specific genes, normalized by an endogenous control gene.

The two pollen-specific genes included in the gene expression analyses were AtPPME1 and CALS5, and the chloroplast-specific genes were cemA, ndhA, and psaA. AtPPME1 is a pectin methylesterase that functions in pollen tube growth; its expression has been found to be

restricted to pollen grains[3,4]. CALS5 is a callose synthase that is highly expressed throughout pollen grain development and is required for pollen exine formation and pollen grain viability[5,6,7]. cemA is a chloroplast envelope membrane protein[8], ndhA is a subunit of NADH dehydrogenase[8], and psaA forms part of the reaction center of photosystem I[8]; all three are encoded by and function within chloroplasts, and the latter two play significant roles in photosynthesis[9-11]. PEX4, a peroxin that enables ubiquitin-protein transferase activity and is expressed in all plant tissues[12], was the endogenous control gene for the RT-PCR experiment.

RNAseq analyses

We analyzed the expression levels of AtPPME1, CALS5, cemA, ndhA, and psaA in the RNAseq data that we generated from *Fragaria chiloensis* and *Raphanus sativus* pollen. The expression levels of the five genes were determined using the nf-core/rnaseq pipeline[13] (https://github.com/nf-core/rnaseq/releases/tag/3.0) and species-specific genome and genome annotation files for *Raphanus sativus* (https://ftp.ncbi.nlm.nih.gov/genomes/all/annotation_releases/3726/100/GCF_000801105.1_Rs1.0/GCF_000801105.1_Rs1.0_genomic.fna.gz, https://ftp.ncbi.nlm.nih.gov/genomes/all/annotation_releases/3726/100/GCF_000801105.1_Rs1.0/GCF_000801105.1_Rs1.0_genomic.gtf.gz). Since a reference genome and genome annotation files for *Fragaria chiloensis* do not currently exist, transcript sequences for AtPPME1, CALS5, cemA, ndhA, and psaA in *Fragaria x ananassa* (a hybrid of *Fragaria chiloensis* and *Fragaria virginiana*) were downloaded from The Genome Database for Rosaceae[14] (https://www.rosaceae.org/Analysis/9642085), and a genome annotation file was manually constructed for the five genes. We used RNAseq data from *Arabidopsis thaliana* leaf tissue

(SRP018034) from the NCBI Short Read Archive (SRA) database (https://trace.ncbi.nlm.nih.gov/Traces/sra/?study=SRP018034)[2] as a standard for cemA, ndhA, and psaA expression and as a negative control for AtPPME1 and CALS5 expression. Specifically, we used fastq reads from two timepoints in *Arabidopsis thaliana* development—early (day 4-1, SRR2079771, GSM1723715: totalRNA4-1; Arabidopsis thaliana; RNA-Seq - SRA - NCBI (nih.gov)) and fully grown (day 16-1, SRR2079777, GSM1723721: totalRNA16-1; Arabidopsis thaliana; RNA-Seq - SRA - NCBI (nih.gov))—and the *Arabidopsis thaliana* genome and genome annotation files from NCBI (https://ftp.ncbi.nlm.nih.gov/genomes/refseq/plant/Arabidopsis_thaliana/latest_assembly_versions/GCF_000001735.4_TAIR10.1/GCF_000001735.4_TAIR10.1_genomic.fna.gz, https://ftp.ncbi.nlm.nih.gov/genomes/refseq/plant/Arabidopsis_thaliana/latest_assembly_versions/GCF_000001735.4_TAIR10.1/GCF_000001735.4_TAIR10.1_genomic.gtf.gz). Exon annotations for cemA, ndhA, and psaA were manually added to the *Raphanus sativus* and *Arabidopsis thaliana* genome annotation files because they were needed for gene quantitation by salmon, a program embedded in the nf-core/rnaseq pipeline[13]. To determine the enrichment of the pollen-specific genes in the RNAseq data from *Fragaria chiloensis* and *Raphanus sativus* pollen and the chloroplast-specific genes in the the RNAseq data from *Arabidopsis thaliana* leaf tissue, ratios between the most highly expressed pollen-specific gene (AtPPME1) and cemA, ndhA, and psaA were calculated using their respective transcripts per million (TPM) values in each RNAseq analysis. To avoid division-by-zero errors when calculating the ratios, a constant (1.0) was added to any TPM values of zero.

We found higher expression (i.e., higher TPM) of AtPPME1 and CALS5 and lower expression of cemA, ndhA, and psaA in the RNAseq data that we generated from *Fragaria*

chiloensis and *Raphanus sativus* pollen (Supplementary Methods Table 1). In contrast, we found higher expression of cemA, ndhA, and psaA and nearly no expression of AtPPME1 and CALS5 in the RNAseq data from *Arabidopsis thaliana* leaf tissue (Supplementary Methods Table 1). We also found that AtPPME1 was 457 to 38,765 times more enriched (i.e., common) than cemA, ndhA, or psaA in the RNAseq data from *Fragaria chiloensis* and *Raphanus sativus* pollen and that the chloroplast-specific genes were 48 to 6,599 times more enriched than AtPPME1 in the RNAseq data from *Arabidopsis thaliana* leaf tissue (Supplementary Methods Figure 1b).

RT-PCR experiment

In 2018, we collected contemporary pollen and leaf tissue from the same *Raphanus sativus* individuals. The pollen was collected and preserved as described above (see "*Pollen collection…"* of the main Methods section). Approximately one hundred leaf discs (500 mg of leaf tissue) spanning the mid-leaf vein were cut using a sterile hole punch, immediately submerged in RNA*later* (Invitrogen, ThermoFisher Scientific, Waltham, MA, USA), and kept at room temperature for seven days until frozen, transported to the University of Pittsburgh (Pittsburgh, PA, USA), and stored at -80ºC. Also as described above, the total RNA was extracted from pollen and at least 50 mg of leaf tissue that was ground into a fine powder in liquid nitrogen. We sent 18 ng of the total RNA from one pollen and one leaf sample to the GRC for RT-PCR with a SYBR green assay type (Power SYBR Green RNA-to C_t 1-Step Kit, Applied Biosystems, ThermoFisher Scientific, Waltham, MA, USA). MacVector (12.7) software (MacVector, Inc., Apex, NC, USA) was used to design custom forward and reverse primers for AtPPME1, CALS5, ndhA, psaA, and PEX4 (Supplementary Methods Table 2), and all primers were purchased from Integrated DNA Technologies, Inc. (Coralville, IA, USA).

We used the double delta C_t method[15] to quantify the relative expression (RT) of AtPPME1, CALS5, ndhA, and psaA in *Raphanus sativus* pollen and leaf RNA. Briefly, we subtracted the average C_t value of the endogenous control gene (PEX4) from the average C_t value of each pollen- or chloroplast-specific gene. The average C_t values were calculated by averaging the C_t values from the three technical RT-PCR replicates of each gene. After multiplying each difference by -1, the resultant values were used as exponents of *e* to calculate the RT of each pollen- or chloroplast-specific gene (Supplementary Methods Table 3). Consistent with the results from the RNAseq analyses, we found that AtPPME1 and CALS5 were relatively highly expressed in *Raphanus sativus* pollen RNA and relatively lowly expressed in *Raphanus sativus* leaf RNA (Supplementary Methods Figure 1ci, ii). In contrast, we found that ndhA and psaA were relatively highly expressed in *Raphanus sativus* leaf RNA and relatively lowly expressed in *Raphanus sativus* pollen RNA (Supplementary Figure 1ciii, iv).

Supplementary Table 1. Plant, floral, and pollen grain traits relevant to life history and interactions with pollinators.

					Pollinator attraction and visitation				Floral reward accessibility		Pollen grain collectability		
**Region[*]**	**Plant subclass, family[†]**	**Plant species and authority**	**Life history[‡]**	**Pollinator functional groups[§]**	**Inflorescence type (size)[‖]**	**Flower longevity (days)[¶]**	**Flower size (mm)[#]**	**Floral rewards[★]**	**Flower symmetry[**]**	**Reward accessibility[††]**	**Pollen grain texture[‡‡]**	**Pollen grain size (μm)[§§]**	**Refs[‖‖]**
CC	*Asteridae, Convolvulaceae*	*Calystegia macrostegia* (Greene) Brummitt	p	ants, bees, beetles	s	1	22 – 68	p, n	r	a	g	85.0	16 – 18
	Caryophyllidae, Aizoaceae	*Carpobrotus edulis* (L.) N. E. Br.	p	bees, beetles, flies	s	3	40 – 90	p, n	r	r	e	37.5	19 – 22
	Magnoliidae, Papaveraceae	*Eschscholzia californica* Cham.	a-p	bees	m (cc)	5	25 – 50	p	r	a	g	46.0	23 – 27
	Rosidae, Rosaceae	*Fragaria chiloensis* (L.) Mill.	p	bees, flies	m (c)	1.5	20 – 40	p, n	r	a	g	30.0	18, 28 – 30
	Magnoliidae, Ranunculaceae	*Ranunculus californicus* Benth.	p	bees	m (c)	11	19	p, n	r	a	e	52.0	25, 31 – 32
	Rosidae, Brassicaceae	*Raphanus sativus* L.	a	bees, flies	m (r)	1.5	50	p, n	r	a	g	32.5	20, 25, 33 – 34
CA	*Magnoliidae, Ranunculaceae*	*Aquilegia canadensis* L.	p	bees, beewasps, birds	m (p)	8	25 – 50	p, n	r	r	e	31.0	35 – 36
	Liliidae, Liliaceae	*Erythronium americanum* Ker Gawl.	p	bees	s	8	25 – 50	p, n	r	a	g	110.0	34, 37
	Asteridae, Asteraceae	*Packera aurea* (L.) Á. Löve & D. Löve	p	bees, flies	m (h)	21	12 – 25	p, n	r	a	e	30.0	22, 25, 38 – 39
	Magnoliidae, Berberidaceae	*Podophyllum peltatum* L.	p	bees	s	6.5	40 – 50	p	r	a	g	42.5	40 – 41
	Rosidae, Saxifragaceae	*Tiarella cordifolia* L.	p	bees, flies	m (r)	16	5	p, n	r	a	g	24.0	40
	Liliidae, Melanthiaceae	*Trillium grandiflorum* (Michx.) Salisb.	p	bees, beetles, flies, wasps	s	14	76 – 102	p, n	r	a	e	54.0	38, 42 – 47
CG	*Liliidae, Liliaceae*	*Calochortus amabilis* Purdy	p	bees, beetles, flies	m (c)	4	25 – 50	p	r	r	g	30.0	25, 48 – 49
	Asteridae, Convolvulaceae	*Calystegia collina* (Greene) Brummitt	p	bees, beewasps	s	1	45	p, n	r	a	g	100.0	16, 50
	Rosidae, Fabaceae	*Cytisus scoparius* (L.) Link	p	bees, beewasps, flies	m (r)	10	20 – 25	p	b	r	g	56.0	51 – 55
	Asteridae, Phrymaceae	*Diplacus aurantiacus* (W. Curtis) Jeps.	p	bees, birds, moths	m (r)	8.5	30 – 50	p, n	b	r	g	60.0	56 – 58
	Liliidae, Iridaceae	*Iris macrosiphon* Torr.	p	bees	s	17.5	70	p, n	b	r	g	125.0	59 – 60
	Rosidae, Fabaceae	*Thermopsis macrophylla* Hook. & Arn.	p	bees	m (r)	10	15 – 20	p, n	b	r	g	23.5	25, 61 – 62
EDAFI	*Asteridae, Convolvulaceae*	*Convolvulus arvensis* L.	p	bees, beewasps, flies	s	1	20 – 25	p, n	r	a	g	67.0	16, 63 – 65
	Asteridae, Balsaminaceae	*Impatiens capensis* Meerb.	a	bees, beewasps, birds	m (c)	4	20 – 25	p, n	b	r	g	36.5	66
	Rosidae, Fabaceae	*Lotus corniculatus* L.	p	bees, beewasps	m (c)	7	10 – 14	p, n	b	r	g	20.0	18, 67 – 68
	Rosidae, Onagraceae	*Oenothera biennis* L.	b	bees, birds, moths	m (r)	1	25	p, n	r	r	g	145.5	25, 38
	Asteridae, Asteraceae	*Solidago* sp. L.	p	bees, beetles, beewasps	m (h)	8	7	p, n	r	a	e	25.0	22, 69
		Vernonia gigantea (Walter) Trel. ex Branner & Coville	p	bees, beeflies, butterflies	m (h)	2	20	p, n	r	a	e	52.5	25, 38, 70

[*]Region: geographic region in which a plant species occurred and from which pollen was collected; CC = California Coast, CA = Central Appalachia, CG = California Grasslands, EDAFI = Eastern Deciduous Agro-forest Interface
[†]Plant subclass, family: subclass and family to which a plant species belongs
[‡]Life history: life cycle (i.e., duration) of a plant species; a = annual, p = perennial, a-p = annual-perennial, b = biennial
[§]Pollinator functional groups: broad taxonomic categories to which the primary pollinators of a plant species belong
[‖]Inflorescence type: type of floral display that a plant species has; s = single-flowered (solitary); m = multiple-flowered (compound cyme [cc], cyme [c], head [h], raceme [r], panicle [p])
[¶]Flower longevity (days): the number of days a flower of a plant species remains open
[#]Flower size (mm): the size of a flower of a plant species across its longest length of attractive tissue
[★]Floral rewards: type of floral rewards a plant species offers to pollinators; p = pollen, n = nectar
[**]Flower symmetry: r = radial, b = bilateral
[††]Reward accessibility: how accessible the floral rewards of a plant species are to pollinators; a = accessible, r = restricted
[‡‡]Pollen grain texture: e = echinate (spiky), g = granulate (non-spiky)
[§§]Pollen grain size (um): diameter of a pollen grain of a plant species across its longest length
[‖‖]Refs: references corresponding to the Supplementary Information Reference list

Supplementary Table 2. The percent contribution of each trait to each PC from the PCA. Only those with a percent contribution of at least 20% were considered significant to a PC.

PC	Trait*	Percent contribution
PC1	Floral symmetry	32.13
	Floral reward accessibility	25.74
	Inflorescence type	20.35
	Flower size	10.95
	Pollen grain texture	6.29
	Flower longevity	1.78
	Rewards	1.77
	Pollen grain size	0.99
PC2	Pollen grain texture	28.48
	Pollen grain size	23.66
	Flower longevity	15.45
	Inflorescence type	13.13
	Flower size	9.33
	Floral reward accessibility	4.68
	Floral symmetry	3.31
	Rewards	1.97
PC3	Flower size	31.47
	Flower longevity	25.74
	Inflorescence type	13.43
	Floral symmetry	10.51
	Floral reward accessibility	8.80
	Pollen grain texture	4.63
	Pollen grain size	3.86
	Rewards	1.56
PC4	Rewards	69.38
	Pollen grain size	21.01
	Pollen grain texture	3.52
	Flower size	2.74
	Floral symmetry	1.60
	Floral reward accessibility	1.37
	Inflorescence type	0.25
	Flower longevity	0.13

*Trait variables in the PCA: inflorescence type; single [0] vs. multiple-flowered [1 = cyme, raceme, panicle, head]), flower longevity (number of days a flower lasts; continuous), flower size (across the longest dimension of a flower, considering all floral tissue; continuous), floral symmetry (zygomorphic [0] vs. actinomorphic [1]), rewards (type of floral rewards available; pollen only [0] vs. pollen and nectar [1]), floral reward accessibility (restricted by morphology or time [0] vs. accessible [1]), pollen grain texture (granulate/smoother/non-spiky [0] vs. echinate/spiky [1]), pollen grain size (across the longest dimension; continuous).

Supplementary Table 3. Sampling, total RNA extraction, total RNA quality check, sequencing, and Pickaxe information for each pollen sample.

Region[*]	Plant species[†]	GPS coordinates[‡]	No. flowers[§]	No. plants[‖]	Lysing time (s)[¶]	A260:A280[#]	Total RNA concentration (ng/μl)[☆]	RNA integrity no.[**]	No. raw reads[††]	No. non-plant reads[‡‡]	No. VRS alignments[§§]	No. quality control contigs[‖‖]	No. viral contigs[¶¶]
CC	*Calystegia macrostegia*	36.0582, -121.5904	6	3	90	1.99	100	5.5	132672932	73524435	704	4599	15
	Carpobrotus edulis	38.3178, -123.0703	3	1	105	2.08	78	9	117859234	89704649	750	12226	2
	Eschscholzia californica	38.3262, -123.008	6	6	90	2.01	47.5	1.9	124923130	3073769	389	213	7
	Fragaria chiloensis	37.5516, -122.5123	7	1	90	1.83	36.9	4	136241938	9346558	187	1261	2
	Ranunculus californicus	38.3162, -123.0685	27	27	90	1.72	33.6	4.9	118632082	83435150	1276	9953	33
	Raphanus sativus	38.3334, -122.97	20	6	105	2.12	76	8.9	122138358	5286039	22944	300	10
CA	*Aquilegia canadensis*	35.6217, -81.5784	10	2	105	2.14	75	8.7	260829738	3843976	440	304	4
	Erythronium americanum	36.1192, -81.8332	12	12	105	2.12	28.2	9.3	249070786	157835111	4	2081	0
	Packera aurea	35.9011, -81.8033	23	11	105	2.07	31.9	8.3	256367020	137249714	8887	11350	37
	Podophyllum peltatum	35.6019, -81.6272	10	10	105	2.1	100	6.4	245181582	163607728	2383	8111	5
	Tiarella cordifolia	35.7311, -81.9031	600	4	105	2.13	96	9.3	243221144	165881525	1498	16363	20
	Trillium grandiflorum	34.9786, -83.4784	5	5	105	2.08	97	2.7	243512428	171104070	212	14701	25
CG	*Calochortus amabilis*	38.866142, -122.453171	18	7	105	2.09	93	5	159151570	130127767	29	24123	3
	Calystegia collina	38.857691, -122.408093	9	9	105	2.11	95	4.8	171348636	71874785	15	2880	11
	Cytisus scoparius	38.8861, -122.5102	13	6	105	2.12	91	6.5	158715652	77405940	1	990	2
	Diplacus aurantiacus	38.8864, -122.5084	6	6	105	2.1	77	6.5	163669076	88694986	15	7885	5
	Iris macrosiphon	38.861049, -122.422534	65	6	105	2.05	97	6.7	151366670	103304665	29	15905	5
	Thermopsis macrophylla	38.859634, -122.411384	20	5	105	2.11	68	4.8	133558876	42217436	58	7413	5
EDAFI	*Convolvulus arvensis*	41.6188, -80.4441	28	3	120	2.14	12.6	6.3	171726922	82228217	21261	1631	28
	Impatiens capensis	41.5734, -80.4974	10	3	120	2.05	39.2	5.6	191278848	172865124	27438120	7409	12
	Lotus corniculatus	41.6188, -80.4441	70	2	120	2.08	93	3.7	195916368	81779531	1777088	331	23
	Oenothera biennis	41.6009, -80.4568	25	5	120	2.09	45.5	3.5	170501670	123463427	1806260	9465	25
	Solidago sp.	41.5734, -80.4974	967	2	120	2.08	29.8	4.1	168296830	113542770	53483	4353	112
	Vernonia gigantea	41.6033, -80.4563	43	3	120	2.13	29.7	4.2	216469370	198115665	187481	5603	47

[*-‡]Region, Plant species, GPS coordinates: geographic area, plant species, and site from which a pollen sample was collected; CC = California Coast, CA = Central Appalachia, CG = California Grasslands, EDAFI = Eastern Deciduous Agro-forest Interface
[§-‖]No. flowers, No. plants: number of flowers and individual plants from which a pollen sample was collected
[¶]Lysing time (s): number of seconds a pollen sample was disrupted using a Qiagen Tissue Lyser II
[#-**]A260:A280, Total RNA concentration (ng/ul), RNA integrity no.: purity ratio, concentration of total RNA extracted, and quality of extracted total RNA as measured by a NanoDrop spectrophotometer, Qubit fluorometer, and via TapeStation analysis at the Genomics Research Core (University of Pittsburgh), respectively
[††]No. raw reads: total number of raw reads obtained from sequencing
[‡‡]No. non-plant reads: number of reads that remained following the Pickaxe subtraction library step
[§§]No. VRS alignments: total number of times the non-plant reads aligned to VRS using Pickaxe
[‖‖]No. quality-control contigs: number of contigs that remained following the Pickaxe contig assembly step and the steps that removed contigs that were too short, heavily masked, or contained highly repetitive sequences
[¶¶]No. viral contigs: number of contigs identified as viral by Pickaxe

Supplementary Table 4. Known viruses identified in the pollen samples by read alignments to VRS. Italicized virus names indicate viruses previously found in association with pollen. NCBI accession numbers indicate the top hit from the alignments to VRS databases. Percent sequence coverage ranges with asterisks represent viruses for which the range began below 20% but ended above 20%, indicating that at least part of a known virus was present. Source data are provided as a source data file.

Virus family*	Virus genus†	Known virus	Region‡	Plant species§	No. segments recovered‖	Percent sequence coverage¶	No. alignments#	NCBI accession nos.
Bromoviridae	*Bromovirus*	Brome mosaic virus	CA<br>EDAFI	*Aquilegia canadensis*<br>*Tiarella cordifolia*<br>*Solidago* sp.	2/3<br>3/3<br>3/3	23.75 – 31.41<br>17.81 – 32.35*<br>19.04 – 41.95*	22 – 29<br>18 – 30<br>10 – 53	NC_002026.1<br>NC_002027.1 NC_002028.2
	Cucumovirus	Peanut stunt virus	EDAFI	*Vernonia gigantea*	1/3	31.31	18	NC_002040.1
	Ilarvirus	Ageratum latent virus	EDAFI	*Impatiens capensis*<br>*Lotus corniculatus*	2/3<br>3/3	9.88 – 22.11*<br>8.86 – 20.02*	22809 – 23257<br>66 – 1122	NC_022127.1 NC_022128.1<br>NC_022129.1
		Apple mosaic virus	CA	*Packera aurea*	2/3	20.61 – 61.58	35 – 174	NC_003465.1 NC_003480.1
		Blackberry chlorotic ringspot virus	EDAFI	*Convolvulus arvensis*<br>*Impatiens capensis*<br>*Lotus corniculatus*<br>*Oenothera biennis*<br>*Solidago* sp.<br>*Vernonia gigantea*	2/3<br>3/3<br>3/3<br>2/3<br>3/3<br>2/3	29.24 – 63.23<br>42.48 – 96.77<br>25.49 – 79.52<br>36.86 – 63.01<br>13.16 – 71.05*<br>26.57 – 57.38	48 – 113<br>470785 – 20653066<br>188 – 344694<br>73 – 347<br>47 – 210<br>47 – 104	NC_011553.1<br>NC_011554.1<br>NC_011555.2
		Parietaria mottle virus	EDAFI	*Impatiens capensis*	2/3	8.16 – 22.66*	42 – 69875	NC_005848.1 NC_005849.1
		Prunus necrotic ringspot virus	CA	*Aquilegia canadensis*<br>*Packera aurea*<br>*Podophyllum peltatum*<br>*Tiarella cordifolia*	1/3<br>3/3<br>2/3<br>3/3	36.08<br>48.50 – 66.99<br>12.93 – 43.28*<br>9.18 – 37.10*	40<br>303 – 5884<br>14 – 71<br>10 – 94	NC_004362.1 NC_004363.1<br>NC_004364.1
		Strawberry necrotic shock virus	EDAFI	*Impatiens capensis*<br>*Lotus corniculatus*	3/3<br>3/3	33.85 – 61.94<br>12.16 – 42.28*	280 – 784607<br>1904 – 257486	NC_008706.1 NC_008707.1<br>NC_008708.2
		Tobacco streak virus	EDAFI	*Lotus corniculatus*<br>*Oenothera biennis*<br>*Solidago* sp.<br>*Vernonia gigantea*	3/3<br>3/3<br>3/3<br>3/3	3.73 – 22.26*<br>96.83 – 99.77<br>98.67 – 99.68<br>16.41 – 51.16*	23 – 151497<br>23169 – 63873<br>10010 – 16311<br>23 – 28	NC_003842.1 NC_003844.1<br>NC003845.1
Partitiviridae	*Alphapartitivirus*	White clover cryptic virus 1	EDAFI	*Solidago* sp.	1/2	54.94	30	NC_006275.1
	Betapartitivirus	White clover cryptic virus 2	EDAFI	*Solidago* sp.	2/2	25.30 – 27.77	18 – 24	NC_021094.1 NC_021095.1
	unclassified	Raphanus sativus cryptic virus 2	CC	*Raphanus sativus*	2/2	99.47 – 100.00	3471 – 6308	NC_010343.1 NC_010344.1
Secoviridae	*Cheravirus*	*Cherry rasp leaf virus*	EDAFI	*Lotus corniculatus*	1/2	20.74	65	NC_006271.1
	Nepovirus	*Tobacco ringspot virus*	EDAFI	*Convolvulus arvensis*<br>*Oenothera biennis*<br>*Solidago* sp.<br>*Vernonia gigantea*	2/2<br>2/2<br>2/2<br>2/2	24.66 – 29.00<br>93.89 – 96.29<br>91.95 – 95.72<br>77.81 – 79.82	102 – 198<br>784429 – 877004<br>1738 – 2460<br>469 – 524	NC_005096.1 NC_005097.1
		Tomato ringspot virus	EDAFI	*Convolvulus arvensis*<br>*Solidago* sp.<br>*Vernonia gigantea*	2/2<br>2/2<br>2/2	39.58 – 45.18<br>39.03 – 49.53<br>26.53 – 35.88	9393 – 10469<br>2227 – 2339<br>265 – 347	NC_003839.2 NC_003840.1
Alphaflexiviridae	*Potexvirus*	White clover mosaic virus	EDAFI	*Solidago* sp.	1/1	71.51	180	NC_003820.1
Iflaviridae	*Iflavirus*	*Deformed wing virus*	EDAFI	*Vernonia gigantea*	1/1	99.53	1711	NC_004830.2
Narnaviridae	*Mitovirus*	Alternaria arborescens mitovirus 1	EDAFI	*Solidago* sp.<br>*Vernonia gigantea*	1/1<br>1/1	55.99<br>22.03	90<br>19	NC_030747.1
		Fusarium globosum mitovirus 1	EDAFI	*Solidago* sp.	1/1	35.79	141	NC_026621.1
No family	unclassified	Hubei narna-like virus 25	EDAFI	*Solidago* sp.	1/1	27.49	17	NC_032727.1
Tombusviridae	*Pelarspovirus*	Pelargonium ringspot virus	CA	*Tiarella cordifolia*	1/1	35.47	274	NC_026240.1
Tymoviridae	*Tymovirus*	Plantago mottle virus	EDAFI	*Convolvulus arvensis*<br>*Solidago* sp.	1/1<br>1/1	34.64<br>61.16	383<br>174	NC_011539.1

*,†Virus family, genus: viral family and genus to which a known virus belongs
‡Region: geographic area in which a known virus was identified; CC = California Coast, CA = Central Appalachia, EDAFI = Eastern Deciduous Agro-forest Interface
§Plant species: plant species in which a known virus was identified
‖No. segments recovered: if the denominator is >1, a known virus has a segmented genome; the numerator denotes how many segments were recovered
¶Percent sequence coverage: the percentage of a top VRS hit covered by our reads; presented as a range if a known virus has a segmented genome; considered present if at least 20%
#No. alignments: the number of times our reads aligned to a top VRS hit; presented as a range if a known virus has a segmented genome; considered present if at least 10

Supplementary Table 5. Novel coding-complete viral genomes and variants of known viruses identified in the pollen samples. The NCBI accession numbers are reflective of the top hit from either BLAST or RAPSearch2 search algorithms. Bolded virus family names show in which families pollen-associated viruses were previously found. Source data are provided as a source data file.

Putative virus family[*]	Putative virus name[†]	Region[‡]	Plant species[§]	No. segments[ǁ]	CD identified[¶]	Contig or extended contig length (nt)[#]	Query coverage[★]	Relative abundance[**]	Nucleotide percent identity[††]	Family-specific percent identity threshold[‡‡]	Algorithm[§§]	NCBI accession nos.
**Bromoviridae**	*Prunus necrotic ringspot virus* (novel variant 1)	CA	*Packera aurea*	3	helicase/ methyltransferase RdRp movement/ coat	1976 - 3314	98.00 – 100.00	0.66 - 9.27	84.78 - 93.37	80% [nucleotide sequences]	blastn/ rapsearch	L38823.1 KT444702.1 JN416774.1
**Partitiviridae**	Ranunculus californicus partitivirus 1	CC	*Ranunculus californicus*	2	RdRp	1890 - 2005	7.63 – 67.62	1.01 – 4.68	42.40 – 49.00	90% (RdRp) or 80% (coat) [amino acid sequences]	rapsearch	ANQ45203.1 ANQ45204.1
	Packera aurea partitivirus 1	CA	*Packera aurea*			1543 - 1582	40.83 – 89.32	69.11 – 584.45	36.20 – 59.40			YP_002364401.1 YP_009255400.1
**Secoviridae**	*Tobacco ringspot virus* (novel variant 1)	EDAFI	*Oenothera biennis*	2	helicase/ RdRp coat	3527 – 7517	99.00	259.76 – 285.17	91.48 – 92.14	80% (protease-RdRp) or 75% (coat) [amino acid sequences]	blastn	KJ556849.1 KJ556850.1
Amalgaviridae	Calystegia macrostegia amalgavirus 1	CC	*Calystegia macrostegia*	1	RdRp	3465	58.96	7.52	59.30	75% [amino acid sequences]	rapsearch	DAB41439.1
Narnaviridae	Fragaria chiloensis narnavirus 1	CC	*Fragaria chiloensis*	1	RdRp	2624	13.15	0.18	39.30	40 – 50% [amino acid sequences]	rapsearch	YP_009408146.1
	Fusarium globosum mitovirus 1 (novel variant 1)	EDAFI	*Solidago* sp.			2364	100.00	0.42	84.54		blastn	LC006128.1
	Solidago narnavirus 1					2445	12.64	0.26	67.00		rapsearch	YP_009272901.1
	Solidago narnavirus 2					2199	11.11	0.64	60.40			YP_009345044.1

[*]Virus family: family to which a novel coding-complete viral genome or variant belongs
[†]Putative virus name: novel coding-complete viral genomes were named after the plant species in which they were identified, as well as the putative viral families to which they belong; novel variants are indicated beneath the name of the known virus
[‡]Region: geographic area in which a novel coding-complete viral genome or variant was discovered; CC = California Coast, CA = Central Appalachia, EDAFI = Eastern Deciduous Agro-forest Interface
[§]Plant species: plant species in which a novel coding-complete viral genome or variant was discovered
[ǁ]No. segments: the number of segments in a novel coding-complete viral genome or variant
[¶]CD identified: the CD(s) bioinformatically identified in a novel coding-complete viral genome or variant
[#]Contig or extended contig length (nt): length of a novel coding-complete viral genome or variant
[★]Query coverage: the percent of a novel coding-complete viral genome or variant that participated in the alignment with the top BLAST or RAPSearch2 hit
[**]Relative abundance: the number of reads assembled into a novel coding-complete viral genome or variant divided by the genome (i.e., contig) length
[††]Nucleotide percent identity: similarity of a novel coding-complete viral genome or variant to the top BLAST or RAPSearch2 hit, where the two align
[‡‡]Family-specific percent identity threshold: novelty assigned based upon ICTV percentage identity criteria for nucleotide or amino acid sequences or specific CDs
[§§]Algorithm: search algorithm used to find similarity between a novel coding-complete genome or variant and the NCBI nucleotide or protein databases

Supplementary Table 6. Novel partial viral genomes and novel partial variants of known viruses identified in the pollen samples. The NCBI accession numbers are reflective of the top hit from either BLAST or Rapsearch2 search algorithms for which a contig was the query. Italicized viral family names indicate viral families previously known to contain pollen-associated viruses. Bolded putative virus names indicate those for which a RdRp conserved domain was recovered, which was included in the relaxed estimate of viral richness. Source data are provided as a source data file.

Putative virus genus, family, or order[*]	Putative virus name[†]	Region[‡]	Plant species[§]	CD identified[‖]	Contig or extended contig length (nt)[¶]	Query coverage[#]	Relative abundance[☆]	Nucleotide percent identity[**]	Family-specific percent identity threshold[††]	Algorithm[‡‡]	NCBI accession nos.
Bromoviridae	*Prunus necrotic ringspot virus* (novel variant 2)	CA	*Aquilegia canadensis*	coat	519	99.00	0.10	95.55	80% [nucleotide sequences]	rapsearch/ blastn	AJ133210.1
	Aquilegia canadensis bromovirus 1			coat	966	67.70	0.35	64.40			AKA64362.1
	Aquilegia canadensis bromovirus 2			movement	693	61.90	0.10	31.50			ABS19899.1
	Aquilegia canadensis bromovirus 3			movement	583	78.22	0.15	60.50			AKA64361.1
	Blackberry chlorotic ringspot virus (novel variant 1)	EDAFI	*Convolvulus arvensis*	movement/coat	1723	100.00	0.21	99.42			JX429883.1
	Grapevine virus S (novel variant 1)			methyltransferase	893	100.00	0.29	99.40			JX513898.1
	***Grapevine virus S* (novel variant 2)**			RdRp	1723	99.00	0.43	99.42			JX513899.1
	Convolvulus arvensis bromovirus 1			coat	794	68.01	0.72	76.20			YP_008470973.1
	Convolvulus arvensis bromovirus 2			helicase	1879	99.82	0.60	72.90			AGN29722.1
	**Convolvulus arvensis bromovirus 3**			RdRp	1319	54.69	0.41	59.20			ALA50795.1
	Blackberry chlorotic ringspot virus (novel variant 2)	EDAFI	*Impatiens capensis*	methyltransferase	1305	99.00	5510.09	95.98			KX834010.1
	***Blackberry chlorotic ringspot virus* (novel variant 3)**			RdRp	2338	99.00	4682.43	94.58			KX834011.1
	***Grapevine virus S* (novel variant 3)**			RdRp	1160	99.00	0.78	99.57			JX513899.1
	Impatiens capensis bromovirus 1			helicase	2742	94.20	0.63	71.30			ACT67442.1
	Impatiens capensis bromovirus 2			movement/ coat	2229	37.28	134.50	70.90			ANN11740.1
	Blackberry chlorotic ringspot virus (novel variant 4)	EDAFI	*Lotus corniculatus*	coat	1477	99.00	25.76	99.04			JX429883.1
	Blackberry chlorotic ringspot virus (novel variant 5)			helicase/ methyltransferase	3115	99.00	2.04	96.13			KX834010.1
	***Blackberry chlorotic ringspot virus* (novel variant 6)**			RdRp	2880	99.00	4.61	94.81			KX834011.1
	Lotus corniculatus bromovirus 1			helicase	1219	99.73	8473.50	71.10			ARS65723.1
	Lotus corniculatus bromovirus 2			movement	1309	63.48	6402.69	70.90			ANN11740.1
	Blackberry chlorotic ringspot virus (novel variant 7)	EDAFI	*Oenothera biennis*	helicase	502	100.00	0.17	95.62		blastn	KX834010.1
	Blackberry chlorotic ringspot virus (novel variant 8)			movement	1133	99.00	0.35	99.65			JX429883.1
	Tobacco streak virus (novel variant 1)			helicase/ methyltransferase	3491	99.00	6.86	99.00			FJ403375.1
	Tobacco streak virus (novel variant 2)			movement/coat	2235	99.00	77.80	98.55			KT445969.1
	***Tobacco streak virus* (novel variant 3)**			RdRp	2947	99.00	12.59	99.18			FJ403376.1
	Apple mosaic virus (novel variant 1)	CA	*Packera aurea*	coat	761	99.00	0.25	94.34		rapsearch/ blastn	AM490197.2
	Packera aurea bromovirus 1			helicase/ methyltransferase	3190	80.63	19.29	56.10			ARO72614.1

Packera aurea bromovirus 2			methyltransferase	501	99.40	0.16	59.30			YP_611154.1
Packera aurea bromovirus 3			movement/ coat	2065	21.65	614.44	38.30			ALO81584.1
**Packera aurea bromovirus 4**			RdRp	2311	55.69	0.59	54.40			ASJ26558.1
**Packera aurea bromovirus 5**			RdRp	2268	56.22	0.38	55.30			ASJ26558.1
**Packera aurea bromovirus 6**			RdRp	2059	64.70	25.13	58.10			NP_619575.1
Prunus necrotic ringspot virus (novel variant 3)	CA	*Podophyllum peltatum*	coat	906	97.00	0.21	93.50		blastn	AJ133207.1
Raphanus latent virus (novel variant 1)	CC	*Raphanus sativus*	helicase/ methyltransferase	3530	66.00	69.24	99.49		rapsearch/ blastn	JN107637.1
Raphanus latent virus (novel variant 2)			movement/coat	2352	57.00	258.04	99.63			JN107639.1
**Raphanus sativus bromovirus 1**			RdRp	2869	62.11	106.16	73.20			ARS65724.1
Ageratum latent virus (novel variant 1)	EDAFI	*Solidago* spp.	helicase	897	80.94	9.57	86.40			YP_008470969.1
Blackberry chlorotic ringspot virus (novel variant 9)			coat	1029	100.00	0.28	99.03			JX429883.1
Blackberry chlorotic ringspot virus (novel variant 10)			methyltransferase	529	98.00	0.09	95.76			KX834010.1
***Grapevine virus S* (novel variant 4)**			RdRp	649	100.00	0.09	99.08			JX513899.1
Tobacco streak virus (novel variant 4)			coat	984	100.00	14.82	97.45			JX073658.1
Tobacco streak virus (novel variant 5)			helicase	631	100.00	3.43	99.37			FJ403375.1
Tobacco streak virus (novel variant 6)			helicase/ methyltransferase	2528	52.33	10.03	81.20			AGW07466.1
Tobacco streak virus (novel variant 7)			helicase/ methyltransferase	2378	100.00	3.42	99.12			FJ403375.1
Tobacco streak virus (novel variant 8)			movement	601	100.00	5.62	94.20			JX463339.1
Tobacco streak virus (novel variant 9)			movement	601	100.00	7.06	99.00			KT445969.1
***Tobacco streak virus* (novel variant 10)**			RdRp	2065	100.00	2.76	99.18			FJ403376.1
***Tobacco streak virus* (novel variant 11)**			RdRp	701	100.00	8.04	91.30			JX463338.1
Solidago bromovirus 1			coat	753	69.32	0.12	76.00			YP_008470973.1
Solidago bromovirus 2			movement/ coat	2184	36.26	12.36	61.20			AFT91205.1
**Solidago bromovirus 3**			RdRp	1404	96.79	6.56	69.50			ACU44509.1
Prunus necrotic ringspot virus (novel variant 4)	CA	*Tiarella cordifolia*	coat	694	97.00	0.27	94.82			JX569825.1
Tiarella cordifolia bromovirus 1			coat	969	67.49	0.57	65.30			AKA64362.1
Tiarella cordifolia bromovirus 2			movement	1020	61.18	0.21	60.10			AKA64361.1
Tiarella cordifolia bromovirus 3			movement	527	96.20	0.45	29.00			ABS19899.1
Blackberry chlorotic ringspot virus (novel variant 11)	EDAFI	*Vernonia gigantea*	movement	580	96.00	0.07	96.26			JX429881.1
***Grapevine virus S* (novel variant 5)**			RdRp	2854	72.00	0.72	99.42			JX513899.1
Vernonia gigantea bromovirus 1			coat	982	67.52	1.01	71.00			YP_008470973.1
Vernonia gigantea bromovirus 2			helicase/ methyltransferase	3467	45.00	0.69	67.70			AQS99321.2

	Vernonia gigantea bromovirus 3			movement/ coat	2271	64.92	0.54	70.90			ANN11740.1
Partitiviridae	**Calystegia macrostegia partitivirus 1**	CC	*Calystegia macrostegia*	RdRp	1632	86.95	10.55	71.00	90% (RdRp) or 80% (coat) [amino acid sequences]	rapsearch/ blastn	AAB27624.1
	**Calystegia macrostegia partitivirus 2**			RdRp	1615	88.42	41.52	71.80			ASU87378.1
	**Cytisus scoparius partitivirus 1**	CG	*Cytisus scoparius*	RdRp	1578	89.54	1.75	58.80			YP_006390091.1
	***Raphanus sativus cryptic virus 2* (novel variant 1)**	CC	*Raphanus sativus*	RdRp	1774	97.00	4.00	96.92			DQ218036.1
	**Solidago partitivirus 1**	EDAFI	*Solidago* spp.	RdRp	2414	15.53	624.40	65.60			YP_003104768.1
	**Solidago partitivirus 2**			RdRp	1761	81.26	302.21	65.50			YP_001686783.1
	**Solidago partitivirus 3**			RdRp	1690	93.30	0.34	84.40			ABV89762.1
	**Vernonia gigantea partitivirus 1**	EDAFI	*Vernonia gigantea*	RdRp	1722	82.58	97.94	67.50			YP_001686783.1
	**Vernonia gigantea partitivirus 2**			RdRp	1699	82.64	157.50	59.60			YP_004429258.1
	**Vernonia gigantea partitivirus 3**			RdRp	724	99.86	0.08	83.80			YP_002308574.1
Secoviridae	*Arracacha virus A* (novel variant 1)	EDAFI	*Convolvulus arvensis*	helicase	1265	99.84	0.15	82.70	80% (protease-RdRp) or 75% (coat) [amino acid sequences]	rapsearch/ blastn	AQW44800.1
	***Arracacha virus A* (novel variant 2)**			RdRp	779	99.74	0.13	79.50			AQW44800.1
	Tomato ringspot virus (novel variant 1)			coat	2003	100.00	9.43	86.76			KR911672.1
	Tomato ringspot virus (novel variant 2)			coat	1576	99.00	6.42	93.80			KR911674.1
	***Tomato ringspot virus* (novel variant 3)**			RdRp	1319	100.00	2.63	98.24			KM083894.1
	Convolvulus arvensis secovirus 1			coat	848	54.48	0.24	63.20			YP_009342469.1
	Convolvulus arvensis secovirus 2			coat	805	99.50	0.19	47.60			YP_009342469.1
	***Grapevine Bulgarian latent virus* (novel variant 1)**	CC	*Eschscholzia californica*	RdRp	3106	66.55	0.32	81.30		rapsearch	YP_004429254.1
	Eschscholzia californica secovirus 1			coat	4851	35.62	1.04	62.80			YP_004429249.1
	Eschscholzia californica secovirus 2			helicase	3346	3.01	0.33	63.60			YP_004429248.1
	Impatiens capensis secovirus 1	EDAFI	*Impatiens capensis*	coat	1334	99.09	0.16	47.60			YP_009342469.1
	Impatiens capensis secovirus 2			helicase	3007	99.75	0.15	70.90			AQW44800.1
	**Impatiens capensis secovirus 3**			RdRp	2099	16.01	0.12	62.50			AQW44800.1
	Lotus corniculatus secovirus 1	EDAFI	*Lotus corniculatus*	coat	1025	37.37	0.11	59.70			YP_009342469.1
	Arracacha virus A (novel variant 3)	EDAFI	*Oenothera biennis*	helicase	1597	99.18	0.15	82.00		rapsearch/ blastn	AQW44800.1
	Tobacco ringspot virus (novel variant 2)			coat	1178	100.00	9.56	87.79			KJ556850.1
	***Tobacco ringspot virus* (novel variant 3)**			RdRp	529	99.00	4.47	85.39			U50869.1
	Oenothera biennis secovirus 1			coat	1330	81.23	0.22	58.80			YP_009342469.1
	**Oenothera biennis secovirus 2**			RdRp	1181	62.76	0.11	71.20		rapsearch	AQW44800.1
	Packera aurea secovirus 1	CA	*Packera aurea*	coat	1648	13.00	0.23	32.39		tblastx	NC_034215.1
	Tobacco ringspot virus (novel variant 4)	EDAFI	*Solidago* spp.	coat	2178	100.00	0.45	91.54		rapsearch/ blastn	KJ556850.1
	Tobacco ringspot virus (novel variant 5)			helicase	3512	99.00	0.40	92.55			U50869.1
	***Tobacco ringspot virus* (novel variant 6)**			helicase/ RdRp	7049	100.00	0.31	94.95			KJ556849.1

	***Tobacco ringspot virus* (novel variant 7)**			RdRp	611	100.00	0.26	96.73			KJ556849.1
	Tomato ringspot virus (novel variant 4)			coat	530	100.00	0.90	87.48			KR911672.1
	Tomato ringspot virus (novel variant 5)			coat	1568	100.00	2.00	92.23			KR911672.1
	***Tomato ringspot virus* (novel variant 6)**			RdRp	953	100.00	0.97	98.46			KR911669.1
	***Tomato ringspot virus* (novel variant 7)**			RdRp	1818	100.00	1.19	98.18			KM083894.1
	Solidago secovirus 1			helicase	890	99.44	0.11	76.60			AQW44800.1
	Tobacco ringspot virus (novel variant 8)	EDAFI	*Vernonia gigantea*	coat	2991	99.00	0.27	93.71			KJ556850.1
	***Tobacco ringspot virus* (novel variant 9)**			helicase/ RdRp	4418	100.00	0.22	93.41			KJ556849.1
	***Tobacco ringspot virus* (novel variant 10)**			RdRp	1096	100.00	0.10	91.67			KJ556849.1
	Tomato ringspot virus (novel variant 8)			coat	4539	100.00	0.30	90.87			KR911670.1
	Tomato ringspot virus (novel variant 9)			helicase	1448	100.00	0.21	92.06			KM083894.1
	***Tomato ringspot virus* (novel variant 10)**			RdRp	2225	100.00	0.17	90.71			KM083894.1
	***Tomato ringspot virus* (novel variant 11)**			RdRp	1173	100.00	0.16	89.46			KR911669.1
	Vernonia gigantea secovirus 1			coat	624	60.10	11280.66	61.90			YP_009342469.1
Alphafleixiviridae	Packera aurea alphaflexivirus 1	CA	*Packera aurea*	coat	512	80.27	0.11	40.00	72% (coat or RdRp) [nucleotide sequences]	rapsearch	ABG88080.1
Amalgaviridae	**Solidago amalgavirus 1**	EDAFI	*Solidago* spp.	RdRp	2373	23.26	2.31	27.20	75% [amino acid sequences]	rapsearch	YP_009388304.1
Aspiviridae	Solidago aspivirus 1	EDAFI	*Solidago* spp.	unknown	620	59.03	0.13	41.80	80% [nucleotide sequences]	rapsearch	BAV13386.1
Betaflexiviridae	***Kalanchoe latent virus* (novel variant 1)**	CC	*Calystegia macrostegia*	RdRp	543	100.00	0.08	86.70	72% (coat or RdRp) [nucleotide sequences]	rapsearch	ACL01040.1
	Calystegia macrostegia betaflexivirus 1			coat	717	28.03	0.09	52.20			CAM12351.1
	Calystegia macrostegia betaflexivirus 2			methyltransferase	1261	99.13	0.08	66.30			AFI61525.1
	***Gaillardia latent virus* (novel variant 1)**	CC	*Ranunculus californicus*	movement/ helicase/ RdRp	1685	51.28	0.19	80.20			YP_009022064.1
	***Verbena latent virus* (novel variant 1)**			RdRp	679	99.41	0.11	85.80			AAF97924.2
	Ranunculus californicus betaflexivirus 1			methyltransferase	782	99.36	0.17	59.50			YP_002302557.1
Caulimoviridae	Calystegia collina caulimovirus 1	CG	*Calystegia collina*	reverse transcriptase/ aspartic protease	1651	41.82	0.22	46.00	80% [nucleotide sequences]	rapsearch	AAO67369.1
	Diplacus aurantiacus caulimovirus 1	CG	*Diplacus aurantiacus*	DNA binding protein	1372	40.08	0.66	42.90			AMN10078.1
	Diplacus aurantiacus caulimovirus 2			movement/ aphid transmission factor	1116	48.39	0.49	68.90			YP_006607888.1
	Diplacus aurantiacus caulimovirus 3			reverse transcriptase/ RNase/ viroplasmin	3665	1.01	0.83	55.60			AMN10080.1
	Iris macrosiphon caulimovirus 1	CG	*Iris macrosiphon*	aspartic protease	3675	9.56	0.50	45.90			NP_569140.1
	Soybean chlorotic mottle virus (novel variant 1)	EDAFI	*Lotus corniculatus*	reverse transcriptase/ RNase	1303	39.83	0.95	82.70			NP_068729.1
	Oenothera biennis caulimovirus 1	EDAFI	*Oenothera biennis*	RNase	819	13.92	0.17	55.30			ABR01170.1

	Thermopsis macrophylla caulimovirus 1	CG	*Thermopsis macrophylla*	RNase	577	59.79	0.49	47.00			YP_009165750.1
	Trillium grandiflorum caulimovirus 1	CA	*Trillium grandiflorum*	viroplasmin	520	24.00	0.19	46.34		tblastx	NC_020999.1
	Yacon necrotic mottle virus (novel variant 1)	EDAFI	*Vernonia gigantea*	reverse transcriptase	611	43.70	0.12	83.10		rapsearch	YP_009121747.1
Chrysoviridae	**Ranunculus californicus chrysovirus 1**	CC	*Ranunculus californicus*	RdRp	3472	91.63	1.44	55.00	80% [nucleotide sequences]	rapsearch	AKU48197.1
Endornaviridae	Trillium grandiflorum endornavirus 1	CA	*Trillium grandiflorum*	helicase	3001	22.89	239.85	34.50	80% [nucleotide sequences]	rapsearch	AFM10600.1
	Trillium grandiflorum endornavirus 2			methyltransferase	1894	31.20	64.63	40.60			YP_009212849.1
	**Trillium grandiflorum endornavirus 3**			RdRp	5713	1.73	159.68	45.80			YP_009212849.1
Geminiviridae	Packera aurea geminivirus 1	CA	*Packera aurea*	movement	647	51.00	0.22	39.10	75% [nucleotide sequences]	rapsearch	AAA46325.1
Idaeovirus (now in Mayoviridae)	Solidago idaeovirus 1	EDAFI	*Solidago* spp.	coat	2234	45.95	3.07	78.60	80% [nucleotide sequences]	rapsearch	CBW59120.1
	Tiarella cordifolia idaeovirus 1	CA	*Tiarella cordifolia*	coat	683	88.73	0.21	67.30			AAZ76537.1
Iflaviridae	**Solidago iflavirus 1**	EDAFI	*Solidago* spp.	RdRp	2518	89.36	3.14	52.90	90% [amino acid sequences]	rapsearch	AKJ70949.1
	Deformed wing virus (novel variant 1)	EDAFI	*Vernonia gigantea*	coat	702	99.00	0.23	99.00		blastn	AY292384.1
	Deformed wing virus (novel variant 2)			coat	1200	100.00	0.16	97.92			AY292384.1
	Deformed wing virus (novel variant 3)			coat	1528	100.00	0.15	98.66			AY292384.1
	Deformed wing virus (novel variant 4)			helicase	5344	100.00	0.20	99.00			AY292384.1
	***Deformed wing virus* (novel variant 5)**			helicase/RdRp	5002	100.00	0.13	99.27			AY292384.1
	***Deformed wing virus* (novel variant 6)**			RdRp	6008	100.00	0.28	99.18			AY292384.1
Luteoviridae	**Lotus corniculatus luteovirus 1**	EDAFI	*Lotus corniculatus*	RdRp	1141	10.52	36.91	70.00	90% [amino acid sequences]	rapsearch	YP_009315896.1
	Lotus corniculatus luteovirus 2			readthrough	683	16.69	29.51	42.10			AFP55338.1
	Lotus corniculatus luteovirus 3			RNA silencing suppressor	723	47.72	58.72	49.60			YP_667839.1
	**Lotus corniculatus luteovirus 4**			Vpg/ RdRp/ coat	1774	41.48	30.15	87.40			YP_009315896.1
	**Solidago luteovirus 1**	EDAFI	*Solidago* spp.	RdRp/ coat	2742	33.70	9.92	71.10			YP_009373263.1
Mononegavirales	**Ranunculus californicus mononegavirales 1**	CC	*Ranunculus californicus*	RdRp	2318	19.31	0.16	34.80	80% [nucleotide sequences]	rapsearch	YP_009304420.1
No family	Calochortus amabilis no family 1	CG	*Calochortus amabilis*	glycosyltransferase	3482	8.00	2.32	28.26	80% [nucleotide sequences]	tblastx	NC_033298.1
	Calystegia collina no family 1	CG	*Calystegia collina*	coat	2008	87.70	0.50	64.60		rapsearch	AHC72012.1
	Calystegia collina no family 2			helicase	1636	62.53	0.30	53.50			AHC72013.1
	Calystegia collina no family 3			helicase	1633	62.65	0.29	53.80			AHC72013.1
	**Calystegia collina no family 4**			RdRp	2836	68.34	0.48	62.40			AHC72013.1
	**Carpobrotus edulis no family 1**	CC	*Carpobrotus edulis*	RdRp	3094	36.75	0.31	68.90			YP_009330120.1
	***Uncultured virus clone 05TGP00448.12* (novel variant 1)**	EDAFI	*Oenothera biennis*	RdRp	762	62.00	0.16	86.68		blastn	JN661368.1
	Packera aurea no family 1	CA	*Packera aurea*	coat	3886	26.25	0.41	53.90		rapsearch	AEM65163.1
	Packera aurea no family 2			helicase	2225	11.00	0.80	39.29		tblastx	NC_035124.1

	Packera aurea no family 3			helicase	1038	81.50	0.25	51.40		rapsearch	AEM65163.1
	Packera aurea no family 4			membrane protein	2184	15.00	2.54	40.00			NC_034152.1
	**Packera aurea no family 5**			RdRp	3579	15.59	2.76	42.50			APG77744.1
	**Packera aurea no family 6**			RdRp	1326	71.95	0.23	57.40			AEM65163.1
	**Ranunculus californicus no family 1**	CC	*Ranunculus californicus*	RdRp	962	99.79	64.32	74.40			YP_009130618.1
	**Ranunculus californicus no family 2**			RdRp	3337	34.07	1.34	68.60			YP_009330120.1
	Ranunculus californicus no family 3			replicase	1883	48.44	0.17	41.40			APG77239.1
	**Raphanus sativus no family 1**	CC	*Raphanus sativus*	RdRp	6779	98.50	2.34	58.90			ASY01343.1
	***Uncultured virus clone 05TGP00448.12* (novel variant 2)**	EDAFI	*Solidago* spp.	RdRp	572	60.00	0.08	87.43		blastn	JN661368.1
	Solidago no family 1			coat	1684	23.57	0.34	43.50		rapsearch	YP_009342462.1
	**Solidago no family 2**			RdRp	2656	77.87	0.15	77.60			YP_009330081.1
	**Solidago no family 3**			RdRp	639	74.18	0.16	55.30			YP_009115495.1
	**Solidago no family 4**			RdRp	1613	50.38	0.44	43.20			YP_009337041.1
	**Solidago no family 5**			RdRp	725	34.53	0.16	43.80			YP_009342464.1
	**Solidago no family 6**			RdRp	726	80.14	0.17	64.20			YP_009330082.1
	**Tiarella cordifolia no family 1**	CA	*Tiarella cordifolia*	RdRp	2311	12.72	0.40	36.70			YP_009336823.1
	**Tiarella cordifolia no family 2**			RdRp	1989	77.22	12.45	68.90			YP_009130618.1
	**Tiarella cordifolia no family 3**			RdRp	709	36.81	0.11	47.10			YP_009336823.1
	Tiarella cordifolia no family 4			serine protease	1340	17.00	0.40	36.84		tblastx	NC_032522.1
	Trillium grandiflorum no family 1	CA	*Trillium grandiflorum*	methyltransferase	2474	28.32	0.29	50.30		rapsearch	YP_009130620.1
	**Trillium grandiflorum no family 2**			RdRp	1724	82.48	7.16	73.80			YP_009026407.1
	**Trillium grandiflorum no family 3**			RdRp	1367	58.60	0.33	40.50			ALD89106.2
	**Trillium grandiflorum no family 4**			RdRp	3444	97.28	0.22	51.70			YP_009130620.1
	**Trillium grandiflorum no family 5**			RdRp	2063	48.17	0.24	40.40			YP_009130620.1
	**Trillium grandiflorum no family 6**			RdRp	510	51.76	0.16	45.50			YP_009182153.1
Ourmiavirus (now in Botourmiaviridae)	**Vernonia gigantea ourmiavirus 1**	EDAFI	*Vernonia gigantea*	RdRp	1748	7.72	0.22	44.40	70% [amino acid sequences]	rapsearch	ALD89131.1
	**Vernonia gigantea ourmiavirus 2**			RdRp	2265	7.68	0.25	39.30			ALD89131.1
Peribunyaviridae	Tiarella cordifolia peribunyavirus 1	CA	*Tiarella cordifolia*	coat	1068	40.45	0.60	37.40	90% [amino acid sequences]	rapsearch	YP_009304992.1
	**Trillium grandiflorum peribunyavirus 1**	CA	*Trillium grandiflorum*	RdRp	1384	25.14	4.43	45.30			ALD89133.1
Phenuiviridae	**Lotus corniculatus phenuivirus 1**	EDAFI	*Lotus corniculatus*	RdRp	7014	10.35	5.55	28.10	80% [nucleotide sequences]	rapsearch	YP_009422199.1
	**Packera aurea phenuivirus 1**	CA	*Packera aurea*	RdRp/ unknown	3802	4.08	0.28	39.00			AHH60917.1
	Raphanus sativus phenuivirus 1	CC	*Raphanus sativus*	coat	3138	33.17	3.71	43.50			YP_009407930.1
	Solidago phenuivirus 1	EDAFI	*Solidago* spp.	coat	966	60.25	2.93	33.70			AOX47532.1
	Solidago phenuivirus 2			glycoprotein	2361	16.26	2.26	38.00			AOX47533.1

	**Solidago phenuivirus 3**			RdRp	3144	47.14	0.60	54.10			AOX47534.1
	Tiarella cordifolia phenuivirus 1	CA	*Tiarella cordifolia*	glycoprotein	1762	56.25	0.18	28.30			NP_941979.1
	Thermopsis macrophylla phenuivirus 1	CG	*Thermopsis macrophylla*	major non-capsid protein	599	68.00	0.10	17.20		tblastx	NC_002328.1
	Lotus corniculatus rhabdovirus 1			methyltransferase	1384	83.88	0.30	57.90			YP_425092.1
	Lotus corniculatus rhabdovirus 2	EDAFI	*Lotus corniculatus*	movement	5470	28.90	0.39	56.40			YP_002308375.1
	**Lotus corniculatus rhabdovirus 3**			RdRp/ methyltransferase	3021	89.28	0.36	59.20			ATS17313.1
Rhabdoviridae	**Lotus corniculatus rhabdovirus 4**			RdRp	1026	99.71	0.27	63.60	80% [nucleotide sequences]	rapsearch	YP_425092.1
	Packera aurea rhabdovirus 1	CA	*Packera aurea*	movement	1194	47.99	0.78	44.00			ATS17310.1
	**Podophyllum peltatum rhabdovirus 1**	CA	*Podophyllum peltatum*	RdRp	1053	27.92	0.35	54.10			YP_002308576.1
	**Trillium grandiflorum rhabdovirus 1**	CA	*Trillium grandiflorum*	RdRp	2988	99.34	0.17	60.60			AFA36170.1
Solemoviridae	**Packera aurea solemovirus 1**	CA	*Packera aurea*	RdRp	1221	41.52	0.46	57.40	80% [nucleotide sequences]	rapsearch	AFP67700.1
	**Solidago tombusvirus 1**	EDAFI	*Solidago* spp.	RdRp/ coat	3302	38.78	0.44	67.50		rapsearch	APA23091.1
Tombusviridae	***Pelargonium ringspot virus*** **(novel variant 1)**	CA	*Tiarella cordifolia*	RdRp/ coat	2396	99.00	0.60	84.13	85% [amino acid sequences]	blastn	AY038068.2
	**Tiarella cordifolia tombusvirus 1**			RdRp	1476	99.00	0.41	82.13			AY038068.2

*Virus genus, family, or order: genus, family, or order to which a novel partial viral genome or variant belongs
†Putative virus name: novel partial viral genomes were named after the plant hosts in which they were identified, as well as the putative viral genus, family, or order to which they belong; where applicable, the numbering scheme continues from Extended Data Table 5, and novel variants are indicated beneath the name of the known virus
‡Region: geographic area in which a novel partial viral genome or variant was discovered; CC = California Coast, CA = Central Appalachia, EDAFI = Eastern Deciduous Agro-forest Interface
§Plant species: plant species in which a novel partial viral genome or variant was discovered
‖CD identified: the CD(s) bioinformatically identified in a novel partial viral genome or variant (i.e., in a contig or extended contig)
¶Contig or extended contig length (nt): length of a novel partial viral genome or variant
#Query coverage: the percent of a novel partial viral genome or variant that participated in the alignment with the top BLAST or RAPSearch2 hit, where the two align
★Relative abundance: the number of reads assembled into a novel partial viral genome or variant divided by the genome (i.e. contig) length
**Nucleotide percent identity: similarity of a novel partial viral genome or variant to the top BLAST or RAPSearch2 hit, where the two align
††Family-specific percent identity threshold: novelty assigned based upon ICTV percentage criteria for nucleotide or amino acid sequences and specific CDs, where applicable
‡‡Algorithm: search program used to find similarity between a novel partial viral genome or variant and the NCBI nucleotide or protein databases

Supplementary Table 7. Plant genomes included in each customized subtraction library.

Region*	Plant species†	Genomes included‡	Genome taxon no. (NCBI)§
CC	*Calystegia macrostegia*	*Ipomoea batatas* (L.) Lam.	4120
	Carpobrotus edulis	*Beta vulgaris* L.	161934
	Eschscholzia californica	*Eschscholzia californica* Cham.	3467
	Fragaria chiloensis	*Fragaria vesca* L.	57918
	Ranunculus californicus	*Aquilegia coerulea* E. James	218851
	Raphanus sativus	*Raphanus raphanistrum* L.	109996
CA	*Aquilegia canadensis*	*Aquilegia coerulea* E. James, *Berberis thunbergii* DC.	11153, 15472
	Erythronium americanum	*Asparagus officinalis* L., *Gastrodia elata* Blume, *Phalaenopsis aphrodite* Rchb. f. *Phalaenopsis equestris* (Schauer) Rchb. f.	10978, 67401, 3206, 11403
	Packera aurea	*Carthamus tinctorius* L., *Erigeron canadensis* L., *Lactuca sativa* L., *Silybum marianum* (L.) Gaertn.	12785, 12828, 352, 40483
	Podophyllum peltatum	*Aquilegia coerulea* E. James, *Berberis thunbergii* DC.	11153, 15472
	Tiarella cordifolia	*Boehmeria nivea* (L.) Gaudich, *Dryas drummondii* Richardson ex Hook., *Fragaria orientalis* Losinsk, *Fragaria nipponica* Makino, *Geum urbanum* L., *Rosa x damascena* Mill., *Rosa multiflora* Thunb., *Ziziphus jujuba* Mill.	14941, 70172, 24460, 24458, 66889, 45184, 11113, 15586
	Trillium grandiflorum	*Asparagus officinalis* L., *Dendrobium officinale* Kimura & Migo, *Phalaenopsis aphrodite* Rchb. f., *Phalaenopsis hybrid cultivar*	10978, 31795, 3206, 34687
CG	*Calochortus amabilis*	*Dendrobium officinale* Kimura & Migo, *Gastrodia elata* Blume, *Phalaenopsis aphrodite* Rchb. f., *Phalaenopsis equestris* (Schauer) Rchb. f.	31795, 67401, 3206, 11403
	Calystegia collina	*Cuscuta australis* R. Br., *Cuscuta campestris* Yunck., *Ipomoea batatas* (L.) Lam., *Ipomoea nil* (L.) Roth, *Ipomoea trifida* (Kunth) G. Don	70252, 69460, 11776, 46552, 37016
	Cytisus scoparius	*Arachis duranensis* Krapov. & W. C. Greg., *Cercis canadensis* L., *Cicer arietinum* L., *Cicer echinospermum* P. H. Davis, *Glycine max* (L.) Merr., *Lotus japonicus* (Regel) K. Larsen, *Nissolia schottii* (Torr.) A. Gray, *Vigna angularis* (Willd.) Ohwi & H. Ohashi	12050, 70179, 2992, 66795, 5, 89, 70174, 11109
	Diplacus aurantiacus	*Dorcoceras hygrometricum* Bunge, *Erythranthe guttata* (Fisch. ex DC.) G. L. Nesom *Fraxinus excelsior* L., *Mentha longifolia* (L.) Huds., *Ocimum tenuiflorum* L., *Olea europaea* L., *Penstemon dissectus* Elliott, *Ruellia speciosa* Mart. ex Nees	12223, 497, 31117, 44852, 40058, 10724, 13465, 50955
	Iris macrosiphon	*Apostasia shenzhenica* Z. J. Liu & L. J. Chen, *Asparagus officinalis* L., *Phalaenopsis aphrodite* Rchb. f., *Phalaenopsis hybrid cultivar*	66931, 10978, 3206, 34687
	Thermopsis macrophylla	*Arachis duranensis* Krapov. & W. C. Greg., *Cajanus cajan* (L.) Millsp., *Cercis canadensis* L., *Glycine max* (L.) Merr., *Lupinus angustifolius* L., *Nissolia schottii* (Torr.) A. Gray, *Phaseolus coccineus* L., *Trifolium pratense* L.	12052, 2878, 70179, 5, 11024, 70174, 10943, 11112
EDAFI	*Convolvulus arvensis*	*Cuscuta australis* R. Br., *Cuscuta campestris* Yunck., *Ipomoea batatas* (L.) Lam., *Ipomoea nil* (L.) Roth, *Ipomoea trifida* (Kunth) G. Don	70252, 69460, 11776, 46552, 37016
	Impatiens capensis	*Embelia ribes* Burm. f., *Monotropa hypopitys* L., *Primula veris* L., *Vaccinium macrocarpon* Aiton	44119, 46551, 35300, 12173
	Lotus corniculatus	*Arachis ipaensis* Krapov. & W. C. Greg, *Cercis canadensis* L., *Cicer echinospermum* P. H. Davis, *Lotus japonicus* (Regel) K. Larsen, *Mucuna pruriens* (L.) DC, *Pisum sativum* L., *Quillaja saponaria* Molina, *Vicia faba* L.	35711, 70179, 66795, 89, 71552, 12050, 71448, 12339
	Oenothera biennis	*Eucalyptus camaldulensis* Dehnh., *Eucalyptus grandis* W. Hill ex Maiden, *Psidium guajava* L.	12405, 2181, 52475
	Solidago sp.	*Cynara cardunculus* var. *scolymus* (L.) Fiori, *Erigeron canadensis* L., *Helianthus annuus* L., *Silybum marianum* (L.) Gaertn.	11286, 12828, 351, 40483
	Vernonia gigantea	*Cynara cardunculus* var. *scolymus* (L.) Fiori, *Lactuca sativa L., Helianthus annuus* L., *Silybum marianum* (L.) Gaertn.	11286, 352, 351, 40483

*,†Region, Plant species: geographic area and plant species from which a pollen sample was collected; CC = California Coast, CA = Central Appalachia, EDAFI = Eastern Deciduous Agro-forest Interface
‡Genomes included: plant genomes included in the customized subtraction library for each pollen sample
§Genome taxon no. (NCBI): taxon number for each genome included in the customized subtraction library for each pollen sample

Supplementary Methods Table 1. TPM of the two pollen-specific genes (AtPPME1 and CALS5) and the three chloroplast-specific genes (cemA, ndhA, and psaA) included in the RNAseq analyses. Source data are provided as a source data file.

**Gene name**	***Fragaria chiloensis***	***Raphanus sativus***	***Arabidopsis thaliana* (early development)**	***Arabidopsis thaliana* (fully grown)**
AtPPME1	975140.8	2237.9	0.0	0.0
CALS5	9332.4	41.7	0.1	0.0
cemA	25.2	4.9	1969.9	47.9
ndhA	1070.9	0.7	453.6	357.0
psaA	888.3	2.0	6141.9	6598.9

Supplementary Methods Table 2. Sequences of the custom forward and reverse primers used to detect expression of AtPPME1, CALS5, ndhA, and psaA in *Raphanus sativus* pollen and leaf RNA using RT-PCR. Source data are provided as a source data file.

Gene name	Primer direction	Primer sequence
AtPPME1	forward	5'- AAGGTTGTCTACGCCTACACCGAG -3'
	reverse	5'- GAACTCTCTTGTCTGTCTGTGCTCC -3'
CALS5	forward	5'- GATGAAGGCTGTAGGAATGTGGG -3'
	reverse	5'- AAACTCGGAGACGAAGGGGAAC -3'
ndhA	forward	5'- CCGATTCAGAGTATGCTCCCATC -3'
	reverse	5'- AGGTTTTTCAGCCCGTCGTG -3'
psaA	forward	5'- TCCACGGTGCTCGTTTTTCC -3'
	reverse	5'- CCCACATCTCCATTCAGGATTTC -3'
PEX4 (endogenous control)	forward	5'- TATTGAAGAACGCCTGGAGCCCTG -3'
	reverse	5'- GGTTTCCTGAGTCGCAGTTGAGAG -3'

Supplementary Methods Table 3. C_t and relative expression values (RT) for AtPPME1, CALS5, ndhA, and psaA in *Raphanus sativus* pollen and leaf RNA. RT expression values were calculated using the double delta method. Source data are provided as a source data file.

Gene name	RNA tissue type	Technical replicate C_t	Average C_t*	ΔC_t†	(-)ΔC_t‡	RT§
AtPPME1	pollen	11.61409<br>12.334153<br>11.999944	11.983	-13.887	13.887	1074348
	leaf	31.154263<br>30.744501<br>30.963072	30.954	4.953	-4.953	0
CALS5	pollen	18.870068<br>19.102768<br>18.923008	18.965	-6.905	6.905	997
	leaf	30.923368<br>30.746893<br>30.638367	30.769	4.769	-4.769	0
ndhA	pollen	23.041977<br>23.416342<br>23.534088	23.331	-2.539	2.539	13
	leaf	21.731632<br>21.500444<br>22.379192	21.870	-4.130	4.130	62
psaA	pollen	23.917028<br>23.883883<br>23.832037	23.878	-1.992	1.992	7
	leaf	17.036053<br>16.941605<br>17.217663	17.065	-8.935	8.935	7595
PEX4 (endogenous control)	pollen	25.597836<br>25.845291<br>26.166733	26.870	na	na	na
	leaf	25.862764<br>26.034098<br>26.104362	26.000			

*Average C_t: calculated by averaging the technical replicate C_t values from either pollen or leaf RNA

†ΔC_t: calculated by subtracting the average PEX4 C_t value from the average C_t value of a gene of interest; the average PEX4 C_t value from pollen RNA was used for all pollen normalizations, and the average PEX4 C_t value from leaf RNA was used for all leaf normalizations; na for the endogenous control gene

‡(-)ΔC_t: calculated by multiplying ΔC_t by -1; na for the endogenous control gene

§RT: calculated by using (-)ΔC_t as the exponent of *e*; na for the endogenous control gene

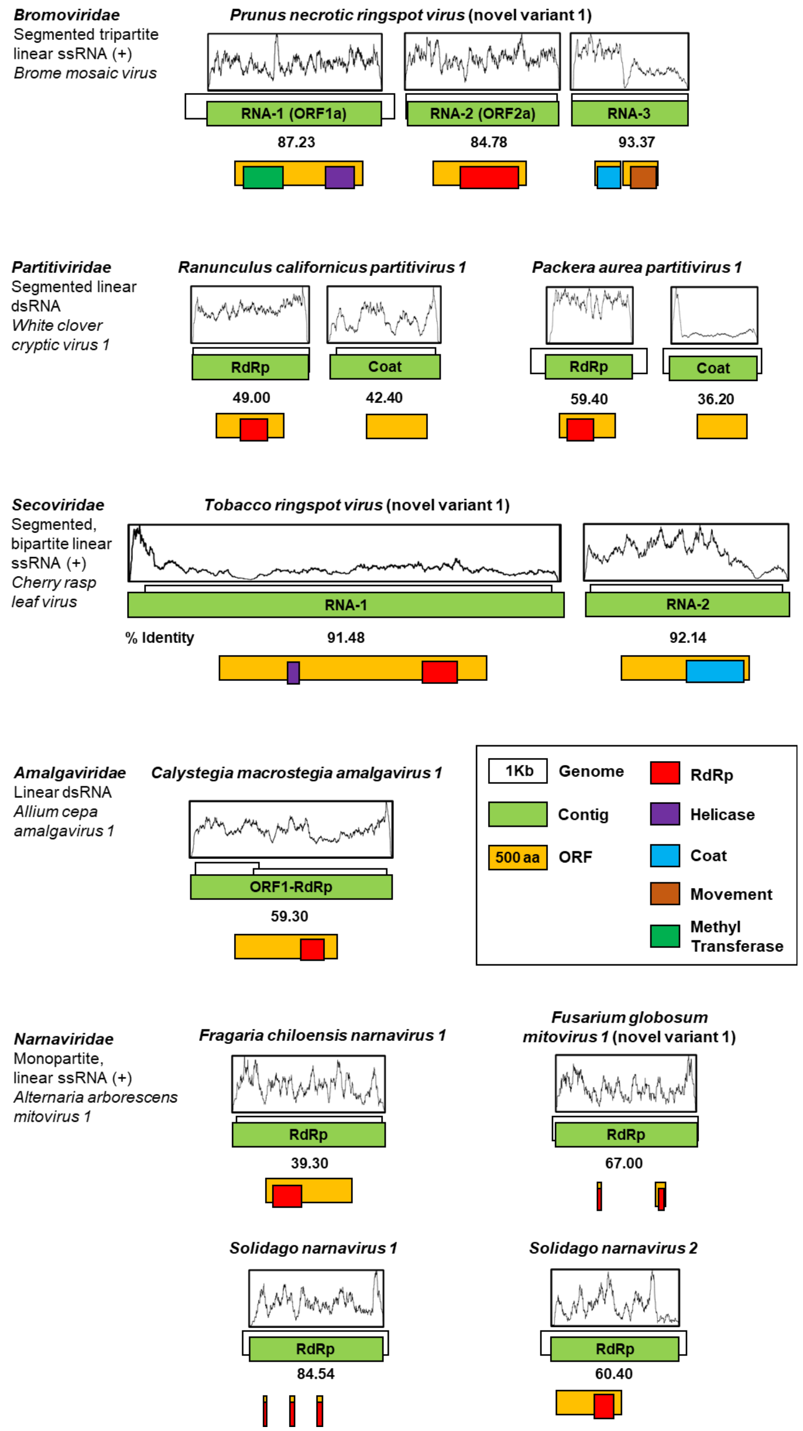

Supplementary Figure 1. Genome organization of the novel coding-complete viral genomes and variants of known viruses. The genome of each virus is shown in comparison to a representative from its putative viral family (below virus family names). Diagrams are drawn to a unified length scale, which is indicated by box length. For each virus, green boxes define contigs, yellow boxes indicate ORFs within contigs, and additional colored boxes refer to specific protein domains (e.g., RdRp, helicase). The numbers below each contig reflect the percent identity to the top BLAST or RAPSearch2 hits as determined by Pickaxe. Coverage plots represent sequencing depth along the genome, where the minimum depth ranges from 0 – 46, and the maximum depth ranges from 30 – 10353. Source data are provided as a source data file.

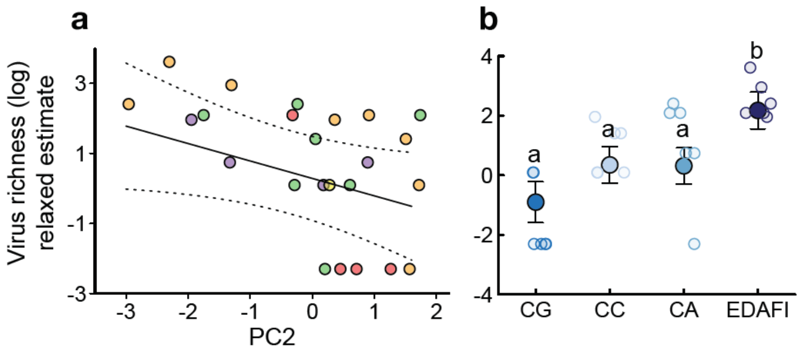

Supplementary Figure 2. Pollen grain traits and region influenced the relaxed estimate of pollen-associated virus richness. (a) Floral PC2 for which lower values reflect spiky and smaller pollen grains, negatively predicted the log-transformed relaxed estimate of virus richness (two-sided chi-squared test, $\chi^2 = 3.73$, df = 1, $P = 0.053$). Colors represent the five subclasses of plant hosts: orange (*Asteridae*), yellow (*Caryophillidae*), purple (*Magnoliidae*), green (*Rosidae*), and red (*Liliidae*), and the dotted lines represent 95% confidence intervals. (b) The log-transformed relaxed estimate of virus richness in each region (two-sided chi-squared test, $\chi^2 = 17.66$, df = 3, $P = 0.0005$). n = 24 biologically independent pollen samples. Error bars represent ±1 standard error. P-values were adjusted using the Tukey method for comparing a family of four estimates. a and b refer to statistically significant differences in the conservative estimate of virus richness between regions. CG = California Grasslands, CC = California Coast, CA = Central Appalachia, EDAFI = Eastern Deciduous Agro-forest Interface. Source data are provided as a source data file.

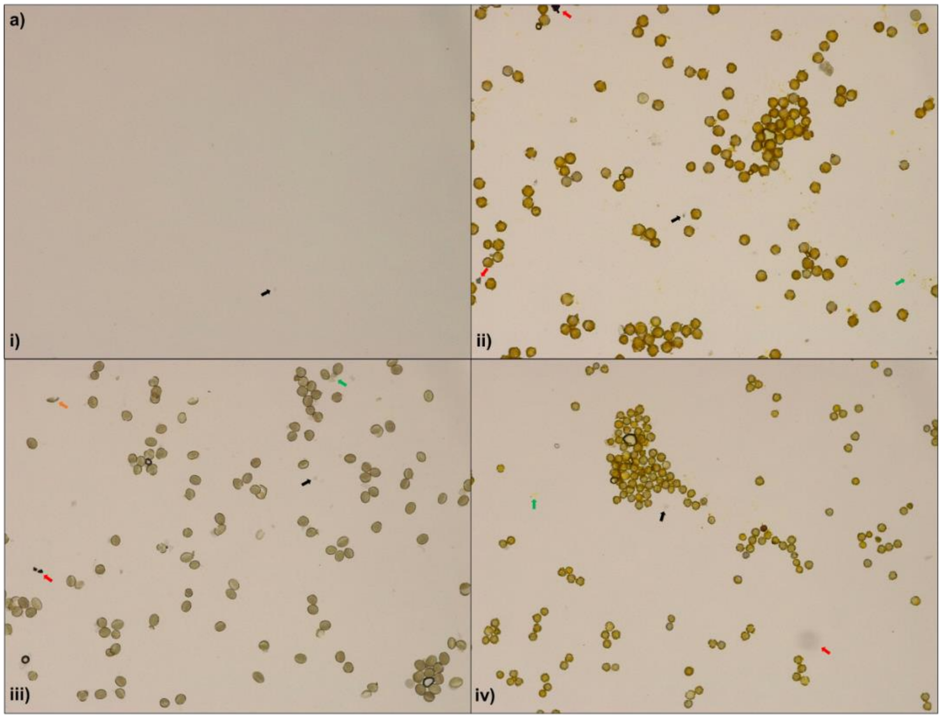

b)	AtPPME1 to chloroplast-specific genes in pollen RNAseq data		Chloroplast-specific genes to AtPPME1 in leaf RNAseq data	
	Fragaria chiloensis	*Raphanus sativus*	*Arabidopsis thaliana* (early development)	*Arabidopsis thaliana* (fully grown)
cemA	38,765	457	1,970	48
ndhA	911	2,999	454	357
psaA	1,098	1,117	6,142	6,599

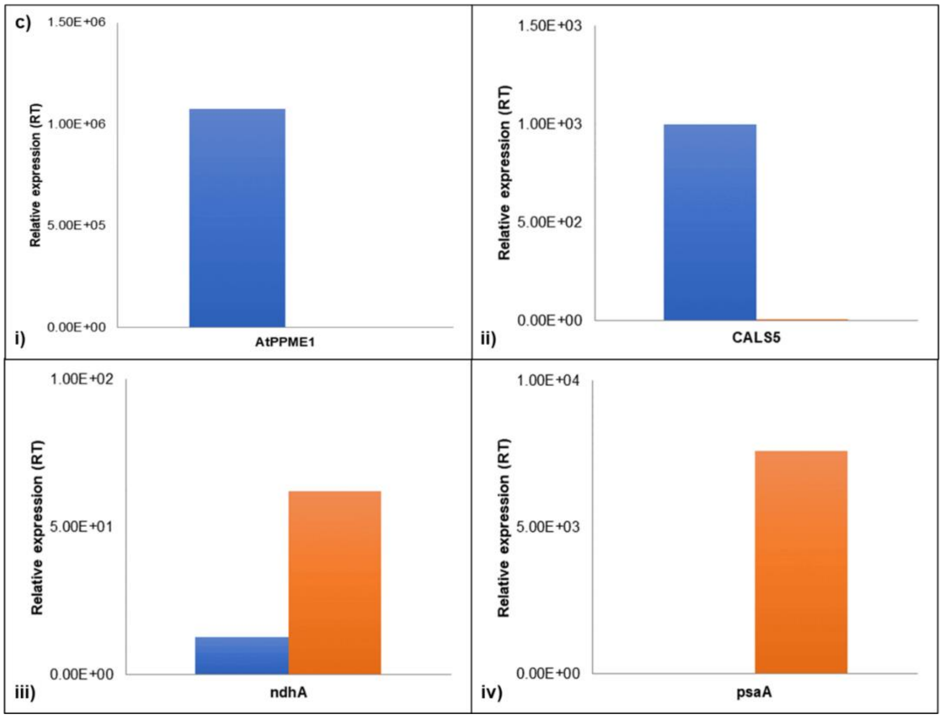

Supplementary Methods Figure 1. (a) Pictures of the control (i) and the *Packera aurea* (ii), *Raphanus sativus* (iii), and *Solidago* sp. (iv) pollen samples, photographed at 10X. Black arrows = examples of background contamination (i.e., dust particles); orange arrow = an example of a pollen exine piece; green arrows = examples of pollen intine or cytoplasm fragments; red arrows = unidentified debris (i.e., potential contamination). (b) Enrichment of AtPPME1 to cemA, ndhA, and psaA in RNAseq data from *Fragaria chiloensis* and *Raphanus sativus* pollen (blue) and enrichment of cemA, ndhA, and psaA to AtPPME1 in RNAseq data from two timepoints in *Arabidopsis thaliana* development (orange) as determined following a single RNAseq analysis. (c) Relative expression values of AtPPME1 (i), CALS5 (ii), ndhA (iii), and psaA (iv) as determined by the double delta C_t method in *Raphanus sativus* pollen (blue bars) and leaf (orange bars) RNA following a single RT-PCR experiment with three technical replicates per gene of interest per tissue type. Source data are provided as a source data file.

Supplementary Information references

1. Birky Jr., C. W., Maruyama, T., Fuerst, P. An approach to population and evolutionary genetic theory for genes in mitochondria and chloroplasts, and some results. *Genetics.* **103,** 513 – 527 (1983).
2. Woo, H. R., Koo, H. J., Kim, J., Jeong, H., Yang, J. O., *et al.* Programming of plant leaf senescence with temporal and inter-organellar coordination of transcriptome in Arabidopsis. *Plant Physiol.* **171,** 452 – 467 (2016).
3. The Arabidopsis Information Resource (TAIR), [AT1G69940(PPME1) (arabidopsis.org)], on www.arabidopsis.org, [14 June 2021].
4. Tian, G.-W., Chen, M.-H., Zaltsman, A., Citovsky, V. Pollen-specific pectin methylesterase involved in pollen tube growth. *Dev. Biol.* **294,** 83 – 91 (2006).
5. Abercrombie, J. M., O'Meara, B. C., Moffatt, A. R., Williams, J. H. Developmental evolution of flowering plant pollen tube cell walls: callose synthase (*CalS*) gene expression patterns. *EvoDevo.* **2,** 14 (2011).
6. Nishikawa, S.-i., Zinkl, G. M., Swanson, R. J., Maruyama, D., Preuss, D. Callose (β-1,3 glucan) is essential for *Arabidopsis* pollen wall patterning, but not tube growth. *BMC Plant Biol.* **5,** 22 (2005).
7. The Arabidopsis Information Resource (TAIR), [AT2G13680(CALS5) (arabidopsis.org)], on www.arabidopsis.org, [14 June 2021].
8. Cheng, H., Li, J., Zhang, H., Cai, B., Gao, Z., *et al.* The complete chloroplast genome sequence of strawberry (*Fragaria x ananassa* Duch.) and comparison with related species of Rosaceae. *PeerJ.* **5,** e3919 (2017).

9. The Arabidopsis Information Resource (TAIR), [AT1G15410 (arabidopsis.org)], on www.arabidopsis.org, [14 June 2021].
10. The Arabidopsis Information Resource (TAIR), [ATCG01100(NDHA) (arabidopsis.org)], on www.arabidopsis.org, [14 June 2021].
11. The Arabidopsis Information Resource (TAIR), [ATCG00350(PSAA) (arabidopsis.org)], on www.arabidopsis.org, [14 June 2021].
12. The Arabidopsis Information Resource (TAIR), [AT5G25760(PEX4) (arabidopsis.org)], on www.arabidopsis.org, [14 June 2021].
13. Ewels, P. A., Peltzer, A., Fillinger, S., Patel, H., Alneberg, J., *et al.* The nf-core framework for community-curated bioinformatics pipelines. *Nat. Biotechnol.* **38,** 276 – 278 (2020).
14. Jung, S., Lee, T., Cheng, C.-H., Buble, K., Zheng, P., *et al.* 15 years of GDR: new data and functionality in the Genome Database for Rosaceae. *Nucleic Acids Res.* **47,** D1137 – D1145 (2019).
15. Hunt, M. Real time PCR. *Microbiology and immunology online. Columbia: Board of Trustees of the University of South Carolina.* (2010).
16. Weaver, S. E., Riley, W. R. The biology of Canadian weeds: 53. *Convolvulus arvensis* L. *Can. J. Plant Sci.* **62**, 461 – 472 (1982).
17. Hanna, C., Naughton, I., Boser, C., Alarcon, R., Hung, K.-L., *et al.* Floral visitation by the Argentine ant reduces bee visitation and plant seed set. *Ecology*. **96**, 222 – 230 (2015).
18. Jepson Flora Project, Jepson eFlora. http://ucjeps.berkeley.edu/eflora/. Accessed 2019.

19. Bartomeus, I., Bosch, J., Vila, M. High invasive pollen transfer, yet low deposition on native stigmas in a *Carpobrotus*-invaded community. *Ann. Bot.* **102**, 417 – 424 (2008).
20. Centre for Agriculture and Bioscience International, Invasive Species Compendium. http://cabi.org/. Accessed 2019
21. Harvard University Herbaria, Flora of North America. http://www.efloras.org. Accessed 2019.
22. Missouri Botanical Garden. http://missouribotanicalgarden.org/. Accessed 2019.
23. Stead, A. D. Pollination-induced flower senescence: a review. *Plant Growth Regul.* **11**, 13 – 20 (1992).
24. Becker, A., Gleissberg, S., Smyth, D. R. Floral and vegetative morphogenesis in California Poppy (*Eschscholzia californica* Cham.). *Int. J. Plant Sci.* **166**, 537 – 555 (2005).
25. Schuh, R. T., Hewson-Smith, S., Ascher, J. S. Discover Life. http://www.discoverlife.org/. Accessed 2019.
26. The American Southwest. https://americansouthwest.net. Accessed 2019.
27. United States Department of Agriculture, Natural Resources Conservation Service, The PLANTS Database. http://plants.usda.gov/. Accessed 2019.
28. Ashman, T.-L., Hitchens, M. S. Dissecting the causes of variation in intra-inflorescence allocation in a sexually polymorphic species, *Fragaria virginiana (Rosaceae). Am. J. Bot.* **87**, 197 – 204 (2000).
29. Penet, L., Collin, C. L., Ashman, T.-L. Florivory increase selfing: an experimental study in the wild strawberry, *Fragaria virginiana. Plant Biol.* **11**, 38 – 45 (2008).

30. Liston, A., Cronn, R., Ashman, T.-L. Fragaria: a genus with deep historical roots and ripe for evolutionary and ecological insights. *Ann. Bot.* **101**, 1686 – 1699 (2014).

31. Dobson, H. Survey of pollen and pollenkit lipids—chemical cues to flower visitors? *Am. J. Bot.* **75**, 170 – 182 (1988).

32. Totland, O. Intraseasonal variation in pollination intensity and seed set in an alpine population of *Ranunculus acris* in Southwestern Norway. *Ecography*. **17**, 159 – 165 (1994).

33. Stanton, M. L. Reproductive biology of petal color variants in wild populations of *Raphanus sativus:* I. Pollinator response to color morphs. *Am. J. Bot.* **74**, 178 – 187 (1987).

34. Lloyd, D. G., Barrett, S. C. H. *Floral Biology: Studies on Floral Evolution in Animal-Pollinated Plants.* (Chapman and Hall, 1996), 420 p.

35. Eckert, C. G., Schaefer, A. Does self-pollination provide reproductive assurance in *Aquilegia canadensis (Ranunculaceae)? Am. J. Bot.* **85**, 919 – 924 (1998).

36. Kliber, A., Eckert, C. G. Sequential decline in allocation among flowers within inflorescences: proximate mechanisms and adaptive significance. *Ecology*. **85,** 1675 – 1687 (2004).

37. Harder, L. D., Thomson, J. D., Cruzan, M. B., Unnasch, R. S. Sexual reproduction and variation in floral morphology in an ephemeral vernal lily, *Erythronium americanum. Oecologia*. **67**, 286 – 291 (1985).

38. Hilty, J. Illinois Wildflowers. http://illinoiswildflowers.info. Accessed 2019.

39. Indiana Native Plant Society, Indiana Native Plants. https://indiananativeplants.org/. Accessed 2019.

40. Motten, A. F. Pollination ecology of the spring wildflower community of a temperature deciduous forest. *Ecol. Monogr.* **56**, 21 – 42 (1986).

41. Whisler, S. L., Snow, A. A. Potential for the loss of self-incompatibility in pollen-limitated populations of mayapple (*Podophyllum peltatum). Am. J. Bot.* **79**, 1273 – 1278 (1992).

42. Kalisz, S., Hanzawa, F. M., Tonsor, S. J., Thiede, D. A., Voight, S. Ant-mediated seed dispersal alters pattern of relatedness in a population of *Trillium grandiflorum. Ecology.* **80**, 2620 – 2634 (1999).

43. Irwin, R. E. Morphological variation and female reproductive success in two sympatric *Trillium* species: evidence for phenotypic selection in *Trillium erectum* and *Trillium grandiflorum* (*Liliaceae). Am. J. Bot.* **87**, 205 – 214 (2000).

44. Griffin, S. R., Barrett, S. C. H. Factors affecting low seed:ovule ratios in a spring woodland herb, *Trillium grandiflorum (Melanthiaceae). Int. J. Plant Sci.* **163**, 581 – 590 (2002).

45. Knight, T. M. Floral density, pollen limitation, and reproductive success in *Trillium grandiflorum. Oecologia.* **137**, 557 – 563 (2003).

46. Knight, T. M. The effects of herbivory and pollen limiation on a declining population of *Trillium grandiflorum. Ecol. Appl.* **14**, 915 – 928 (2004).

47. Schmucki, R., de Blois, S. Pollination and reproduction of a self-incompatible forest herb in hedgerow corridors and forest patches. *Oecologia.* **160**, 721 – 733 (2009).

48. Holtsford, T. P. Nonfruiting hermaphroditic flowers of *Calochortus leichtlinii (Liliaceae)*: potential reproductive functions. *Am. J. Bot.* **72**, 1687 – 1694 (1985).

49. Dilley, J. D., Wilson, P., Mesler, M. R. The radiation of *Calochortus:* generalist flowers moving through a mosaic of potential pollinators. *Oikos.* **89**, 209 – 222 (2000).

50. Wolf, A. T., Harrison, S. Natural habitat patchiness affects reproductive success of serpentine morning glory (*Calystegia collina*, *Convolvulaceae*). *Conserv. Biol.* **15**, 111 – 121 (2001).

51. Parker, I. M. Pollinator limitation of *Cytisus scoparius* (Scotch broom), an invasive exotic shrub. *Ecology.* **78**, 1457 – 1470 (1997).

52. Parker, I. M., Haubensak, K. A. Comparative pollinator limitation of two non-native shrubs: do mutualisms influence invasions? *Oecologia.* **130**, 250 – 258 (2002).

53. Simpson, S. R., Gross, C. L., Silberbauer, L. X. Broom and honeybees in Australia: an alien liaison. *Plant Biol.* **7**, 541 – 548 (2005).

54. Paynter, Q., Main, A., Gourlay, A. H., Peterson, P. G., Fowler, S. V., *et al.* Disruption of an exotic mutualism can improve management of an invasive plant: varroa mite, honeybees, and biological control of Scotch broom *Cytisus scoparius* in New Zealand. *J. Appl. Ecol.* **47**, 309 – 317 (2010).

55. Muir, J. L., Vamosi, J. C. Invasive Scotch broom (*Cytisus scoparius*, *Fabaceae*) and the pollination success of three Garry oak-associated plant species. *Biol. Invasions.* **17**, 2429 – 2446 (2010).

56. Baldwin, B. G., Boyd, S., Ertter, B., Patterson, R., Rosatti, T. J., et al. *The Jepson Desert Manual: Vascular Plants of Southeastern California* (University of California Press, 2002), 640 p.

57. Streisfeld, M. A., Kohn, J. R. Environment and pollinator-mediated selection on parapatric floral races of *Mimulus aurantiacus. J. Evol. Biol.* **20**, 122 – 132 (2006).

58. Peay, K. G., Belisle, M., Fukami, T. Phylogenetic relatedness predicts priority effects in nectar yeast communities. *Proc. R. Soc. B.* **279**, 749 – 758 (2012).
59. Garden Design for Living. http://gardendesignforliving.com. Accessed 2019.
60. Pacific Bulb Society. http://pacificbulbsociety.org/. Accessed 2019.
61. Gori, D. F. Floral color change in *Lupinus argenteus (Fabaceae)*: why should plants advertise the location of unrewarding flowers to pollinators? *Evolution.* **43**, 870 – 881 (1989).
62. Pollinator Partnership. http://pollinator.org/. Accessed 2019.
63. Harmon-Threatt, A. N., Burns, J. H., Shemyakina, L. A., Knight, T. M. Breeding system and pollination ecology of introduced plants compared to their native relatives. *Am. J. Bot.* **96**, 1544 – 1550 (2009).
64. Sonday, B., Bradtke, J., Burnham, R. J. Climbers: censusing lianas in mesic biomes of Eastern regions: *Convolvulus arvensis*. http://climbers.lsa.umich.edu/?p=215. Accessed 2019.
65. Prokop, P., Neupauerova, D. Flower closure in the field bindweed (*Convolvulus arvensis*): a field test of the pollination hypothesis. *Turk. J. Bot.* **38**, 877 – 882 (2014).
66. Steets, J. A., Hamrick, J. L., Ashman, T.-L. Consequences of vegetative herbivory for maintenance of intermediate outcrossing in an annual plant. *Ecology*. **87**, 2717 – 2727 (2006).
67. Hegland, S. J., Totland, O. Is the magnitude of pollen limitation in a plant community affected by pollinator visitation and plant species specialization levels? *Oikos.* **117**, 883 – 891 (2008).

68. Gao, J., Xiong, Y.-Z., Huang, S.-Q. Effects of floral sexual investment and dichogamy on floral longevity. *J. Plant Ecol.* **8**, 116 – 121 (2014).

69. Gross, R. S., Werner, P. A. Relationships among flowering phenology, insect visitors, and seed-set of individuals: experimental studies on four co-occurring species of goldenrod (*Solidago; Compositae*). *Ecol. Monogr.* **53**, 95 – 117 (1983).

70. Rao, M. M., Raju, A. J. S., Ramana, K. V. Secondary pollen presentation and psycholphily in *Vernonia albicans* and *V. cinereal (Asteraceae). Phytol. Balc.* **23**, 171 – 186 (2017).
