# Supplementary material for: The pollen virome of wild plants and its association with variation in floral traits and land use
Source: Nat Commun. 2022 Jan 26;13:523. doi: 10.1038/s41467-022-28143-9 (PMC8791949; doi:10.1038/s41467-022-28143-9)
Supplement: Supplementary file 3 — Description of Additional Supplementary Information [file 41467_2022_28143_MOESM3_ESM.pdf]

Description of Additional Supplementary Information

Title: Supplementary Dataset 1

Description: Pickaxe Virus RefSeq alignments. This dataset includes all Pickaxe output from the viral read alignments to VRS. It also reports whether each met our alignment (at least 10) and percent sequence coverage (at least 20%) criteria to denote the presence of a known virus, and whether each represents a segment or entire genome.

Title: Supplementary Dataset 2

Description: Pickaxe contig alignments. This dataset includes all Pickaxe output from the viral contig alignments to NCBI nucleotide and protein databases and all the viral contig sequences. It also reports the open reading frame and conserved domain annotation for each contig and whether each represents a novel coding-complete viral genome or variant or a novel partial viral genome or variant.
