# Supplementary material for: The pollen virome of wild plants and its association with variation in floral traits and land use
Source: Nat Commun. 2022 Jan 26;13:523. doi: 10.1038/s41467-022-28143-9 (PMC8791949; doi:10.1038/s41467-022-28143-9)
Supplement: Supplementary file 6 — Reporting Summary [file 41467_2022_28143_MOESM6_ESM.pdf]

nature research

Corresponding author(s): Andrea M. Fetters and Tia-Lynn Ashman, NCOMMS-20-45412C

Last updated by author(s): Dec 15, 2021

Reporting Summary

Nature Research wishes to improve the reproducibility of the work that we publish. This form provides structure for consistency and transparency in reporting. For further information on Nature Research policies, see our Editorial Policies and the Editorial Policy Checklist.

Statistics

For all statistical analyses, confirm that the following items are present in the figure legend, table legend, main text, or Methods section.

n/a	Confirmed	
☐	☒	The exact sample size (*n*) for each experimental group/condition, given as a discrete number and unit of measurement
☐	☒	A statement on whether measurements were taken from distinct samples or whether the same sample was measured repeatedly
☐	☒	The statistical test(s) used AND whether they are one- or two-sided<br>*Only common tests should be described solely by name; describe more complex techniques in the Methods section.*
☐	☒	A description of all covariates tested
☐	☒	A description of any assumptions or corrections, such as tests of normality and adjustment for multiple comparisons
☐	☒	A full description of the statistical parameters including central tendency (e.g. means) or other basic estimates (e.g. regression coefficient) AND variation (e.g. standard deviation) or associated estimates of uncertainty (e.g. confidence intervals)
☐	☒	For null hypothesis testing, the test statistic (e.g. *F*, *t*, *r*) with confidence intervals, effect sizes, degrees of freedom and *P* value noted<br>*Give P values as exact values whenever suitable.*
☒	☐	For Bayesian analysis, information on the choice of priors and Markov chain Monte Carlo settings
☒	☐	For hierarchical and complex designs, identification of the appropriate level for tests and full reporting of outcomes
☐	☒	Estimates of effect sizes (e.g. Cohen's *d*, Pearson's *r*), indicating how they were calculated

Our web collection on statistics for biologists contains articles on many of the points above.

Software and code

Policy information about availability of computer code

Data collection

We used a viral discovery pipeline, Pickaxe, to identify known viruses and novel viral genomes in our pollen samples. The code for this unique pipeline is available upon request and can be accessed at https://github.com/pcantalupo/pickaxe or at Zenodo: doi: 10.5281/zenodo.5718362. Pickaxe used the Bowtie2 aligner (v2.3.4.2-3) to remove host or contaminant genomes downloaded from the National Center for Biotechnology Information (NCBI) database. It also used Bowtie2 to align viral non-host reads to Viral RefSeq (NCBI, https://ftp.ncbi.nlm.nih.gov/refseq/release/viral/). In addition, viral reads were assembed into contigs using the CLC Assembly Cell (Qiagen Digital Insights, Redwood City, CA, USA). Pickaxe was then used to remove repetitive, short, and heavily masked contig sequences. Contigs that passed these quality control measures were aligned to GenBank nucleotide and protein databases using BLAST (NCBI) and Rapsearch2 (v2.22) search algorithms. Open reading frames (ORFs) and viral conserved domains (CDs) were found in the contigs using ORFfinder (NCBI, default parameters, https://www.ncbi.nlm.nih.gov/orffinder/, v0.4.3), and by searching the Conserved Domain Database (NCBI, default parameters, https://www.ncbi.nlm.nih.gov/Structure/cdd/wrpsb.cgi, v3.16-17), respectively. Depth plots showing the coverage across all novel coding-complete viral genomes were created using Bowtie2 and Samtools (v1.9).

We constructed the phylogeny of the host plant species in R (v4.0.1) and the family-level viral phylogenies using MEGA X. All R packages used are reported in the manuscript and in the "Data Analysis" section of the "Software and Code" section of the Reporting Summary.

We used ArcGIS Desktop (v10.7.1) and the National Geospatial Data Asset (NGDA) Land Use Land Cover dataset (v2014) to characterize the land use for each region.

We used a Leica ICC50 W Camera (Module and Firmware versions 2016.1.0.6995 and 1.30.391676, respectively) to take pictures of pollen samples to assess their lack of contamination at the microscopic level.

We determined the expression levels of two pollen-specific genes (AtPPME1, CALS5) and three chloroplast specific genes (cemA, ndhA, psaA) in the RNAseq data that we generated from two pollen samples (Raphanus sativus and Fragaria chiloensis) using the nf-core/rnaseq pipeline

	(https://github.com/nf-core/rnaseq/releases/tag/3.0) and species-specific genome and genome annotation files for Raphanus sativus (https://ftp.ncbi.nlm.nih.gov/genomes/all/annotation_releases/3726/100/GCF_000801105.1_Rs1.0/GCF_000801105.1_Rs1.0_genomic.fna.gz, https://ftp.ncbi.nlm.nih.gov/genomes/all/annotation_releases/3726/100/GCF_000801105.1_Rs1.0/GCF_000801105.1_Rs1.0_genomic.gtf.gz). Fragaria transcripts were downloaded from The Genome Database for Rosaceae (https://www.rosaceae.org/Analysis/9642085). We used RNAseq data from Arabidopsis thaliana leaves (SRP018034) from the NCBI Short Read Archive (SRA) database (https://trace.ncbi.nlm.nih.gov/Traces/sra/?study=SRP018034) as a standard for cemA, ndhA, and psaA expression and as a negative control for AtPPME1 and CALS5 expression. Specifically, we used fastq reads from two timepoints in Arabidopsis thaliana development—early (day 4-1, SRR2079771, GSM1723715: totalRNA4-1; Arabidopsis thaliana; RNA-Seq - SRA - NCBI (nih.gov)) and fully grown (day 16-1, SRR2079777, GSM1723721: totalRNA16-1; Arabidopsis thaliana; RNA-Seq - SRA - NCBI (nih.gov))—and the Arabidopsis thaliana genome and genome annotation files from NCBI (https://ftp.ncbi.nlm.nih.gov/genomes/refseq/plant/Arabidopsis_thaliana/latest_assembly_versions/GCF_000001735.4_TAIR10.1/GCF_000001735.4_TAIR10.1_genomic.fna.gz, https://ftp.ncbi.nlm.nih.gov/genomes/refseq/plant/Arabidopsis_thaliana/latest_assembly_versions/GCF_000001735.4_TAIR10.1/GCF_000001735.4_TAIR10.1_genomic.gtf.gz). Gene quantitation was performed with salmon, a program embedded in the nf-core/rnaseq pipeline.<br><br>We designed custom forward and reverse RT-PCR primers for two pollen-specific genes (AtPPME1, CALS5), two chloroplast-specific genes (psaA, ndhA), and one endogenous control gene (PEX4) in Raphanus sativus using MacVector (v12.7) software (MacVector, Inc., Apex, NC, USA).
Data analysis	All statistical analyses were performed in R (v4.0.1). R packages used were: "ape" (v5.0), "phytools" (v0.7-47), "vegan" (v2.5-6), "gplots" (v3.0.3), "Heatplus" (v2.34.0), RColorBrewer" (v1.1-2), "FactoMineR" (v2.4), "nlme" (v3.1-148), "car" (v3.0-9), and "emmeans" (v1.4.7). Any R code generated for standard data analysis is available upon request.

For manuscripts utilizing custom algorithms or software that are central to the research but not yet described in published literature, software must be made available to editors and reviewers. We strongly encourage code deposition in a community repository (e.g. GitHub). See the Nature Research guidelines for submitting code & software for further information.

Data

Policy information about availability of data

All manuscripts must include a data availability statement. This statement should provide the following information, where applicable:

- Accession codes, unique identifiers, or web links for publicly available datasets
- A list of figures that have associated raw data
- A description of any restrictions on data availability

The raw reads were deposited in GenBank under Bioproject number PRJNA589022 and will be publicly available upon publication. The Pickaxe output from viral read alignments to VRS and the Pickaxe output from viral contig alignments to the GenBank nucleotide and protein databases are included as Supplementary Datasets 1 and 2, respectively. All contig sequences are also included in Supplementary Dataset 2. In addition, supplementary information and source data are provided with this paper.

All other data used for this manuscript are reported in the "Data Collection" section of the "Software and Code" section of the Reporting Summary in junction with the software used to analyze it and are reported similarly in the "Methods" and "Supplementary Methods" sections of the paper.

Field-specific reporting

Please select the one below that is the best fit for your research. If you are not sure, read the appropriate sections before making your selection.

☐ Life sciences ☐ Behavioural & social sciences ☒ Ecological, evolutionary & environmental sciences

For a reference copy of the document with all sections, see nature.com/documents/nr-reporting-summary-flat.pdf

Ecological, evolutionary & environmental sciences study design

All studies must disclose on these points even when the disclosure is negative.

Study description	To uncover the diversity of pollen-associated viruses, and understand landscape and floral features that drive pollen-mediated viral spread, we performed a species-level metagenomic survey of pollen from wild, asymptomatic plants (24 species, 16 families, five subclasses), located in one of four regions (California Grasslands, California Coast, Central Appalachia, Eastern Deciduous Agro-forest Interface) in the United States that vary in human land use.
Research sample	As we were interested in identifying known viruses and novel viral genomes and variants associated with pollen (i.e., located on the outside or inside of pollen grains), we collected pollen samples (30 - 50 mg) from 24 plant species. The plant species were: Calystegia collina, Calochortus amabilis, Cytisus scoparius, Diplacus aurantiacus, Iris macrosiphon, Thermopsis macrophylla (California Grasslands), Convolvulus arvensis, Impatiens capensis, Lotus corniculatus, Oenothera biennis, Solidago sp., Vernonia gigantea (Eastern Deciduous Agro-forest Interface), Calystegia macrostegia, Carpobrotus edulis, Eschscholzia californica, Fragaria chiloensis, Ranunculus californica, Raphanus sativus (California Coast), Aquilegia canadensis, Erythronium americanum, Podophyllum peltatum, Packera aurea, Tiarella cordifolia, and Trillium grandiflorum (Central Appalachia). The pollen samples were meant to represent the local populations in the specific locations of the four regions (reported below in the "Location" section of the "Field work, Collection, and Transport" section of the Reporting Summary) from which they were collected.<br><br>In addition, we collected one hundred leaf discs (500 mg of leaf tissue) from the same Raphanus sativus individuals from which we collected pollen, at the time of pollen collection.

No manipulations were made.

Sampling strategy

At each of the four regions, we identified visually asymptomatic individuals of wild plant species that were in full flower and in high enough abundance to achieve our pollen sample minimum (30 mg). To achieve the broadest representation of plant species, we selected species in different families when possible. We focused mainly on perennial species to avoid any effects of life-history variation. From these, we collected 30 to 50 mg of pollen from newly dehiscing anthers in situ using a sterile sonic dismembrator (Fisherbrand Model 50, Fisher Scientific, Waltham, MA, USA) with a frequency of 20 Hz. We removed non-pollen tissues (e.g., anther debris) with sterile forceps. Visibly pure pollen from a single species was transferred to a 2-mL collection tube with Lysing Matrix D (MP Biomedicals, Irvine, CA, USA) and kept on dry ice until transported to and stored at -80ºC at the University of Pittsburgh. Statistical methods were not used to predetermine sample size; rather, we conducted RNA extraction trials on varying amounts of pollen collected from flowers available in the University of Pittsburgh's greenhouse, as detailed for this project, before we collected pollen for this project. Through the trials, we aimed to find a volume of pollen from which we could consistently extract enough high quality RNA for subsequent sequencing.

We collected one hundred leaf discs (500 mg of leaf tissue) using a sterile hole punch from the same Raphanus sativus individuals from which we collected pollen. All the leaf discs spanned the the mid-leaf vein and were immediately submerged in RNAlater (Invitrogen, ThermoFisher Scientific, Waltham, MA, USA) and kept at room temperature for seven days until frozen, transported to the University of Pittsburgh, and stored at -80ºC. The amount of leaf tissue collected followed the manufacturer's recommendation in the Quick-RNA Plant Miniprep Extraction Kit (Zymo Research Corporation, Irvine, CA, USA), which we used to extract RNA throughout the project.

Data collection

Pollen sampling data for each plant species included the number of flowers and plants from which pollen was collected and the GPS coordinates of collection sites within each of the regions (see the "Location" section of the of the "Field work, Collection, and Transport" section of the Reporting Summary). Additional plant traits were scored from the literature, and land use in each region was calculated using GIS technology (see the "Data Collection" section of the "Software and Code" section of the Reporting Summary). All except the land use was recorded using pen and paper. The GPS coordinates were determined using the "Maps" app on an iPhone.

Raphanus sativus leaf sampling data included the number of plants from which the tissue was collected (the same 18 individuals from which we collected pollen), the GPS coordinates of collection sites within each of the regions (see the "Location" section of the of the "Field work, Collection, and Transport" section of the Reporting Summary), and how the tissue was preserved and stored (see the "Sampling Strategy" section of the "Ecological, Evolutionary, and Environmental Studies Design" section of the Reporting Summary). This data was recorded using pen and paper. The GPS coordinates were determined using the "Maps" app on an iPhone.

Pollen RNA extraction data included pollen grain size and texture, how long a pollen sample was lysed, and the concentration, A260:A280 purity ratio, and RNA integrity value of the total RNA extracted from a pollen sample. The Raphanus sativus leaf extraction data included how the tissue was lysed (in liquid nitrogen), and the concentration, A260:A280 purity ratio, and RNA integrity value of the total RNA extracted. Data was recorded using pen and paper. The concentrations were measured using a Qubit 2.0 fluorometer (Invitrogen, ThermoFisher Scientific, Waltham, MA, USA), the purity ratios were measured using a NanoDrop spectrophotometer (ThermoFisher Scientific< Waltham, MA, USA), and the RNA integrity values were measured by the Genomics Research Core (GRC) at the University of Pittsburgh via TapeStation analysis.

Only RNA from pollen was sequenced. Next-generation sequencing data included the number of raw reads, recorded into Excel spreadsheets. Pickaxe output/data, deposited into Excel spreadsheets, included the number of non-host reads, number of read alignments to Virus RefSeq (NCBI), number of contigs that passed the quality control steps, and the number of viral contigs. Other relevant Pickaxe output/data, deposited into Excel spreadsheets, also included the length of a contig, the top hit from viral non-host read or contig alignments to Virus RefSeq (NCBI) or GenBank protein or nucleotide databases (NCBI), how similar our viral non-host read or contig was to a reference genome (percent identity), and how much our viral non-host read or contig covers a reference genome (percent sequence coverage or query coverage, respectively). The open reading frames and conserved domains found in a contig, as well as their stop/start positions within a genome and lengths, were recorded into Excel spreadsheets after using ORFfinder (NCBI) and searching the Conserved Domain Database (NCBI), as described above in the "Data Collection" section of the "Software and Code" section of the Reporting Summary. The conservative and relaxed estimates of virus richness were calculated in Excel by tallying the known viruses and novel coding-complete genomes and variants (conservative) or adding the novel partial genomes and variants (RdRps only) to the conservative estimate of virus richness (relaxed) in Excel.

The representative plant species used for the evaluations of pollen sample purity detailed in the Supplementary Methods were chosen because they had either relatively low or relatively high estimates of pollen-associated virus richness. The data in the microscopy analysis were recorded using pen and paper and included the counts of intact pollen grains, pollen grain exine, intine, or cytoplasm fragments, debris similar to that seen in the control (e.g., dust particles), and unidentified debris (i.e., potential contaminants) in three aliquots of pollen samples from Packera aurea, Raphanus sativus, and the Solidago sp., or in three aliquots from a control. Calculations were done in Excel. The data from the RNAseq analysis on our trimmed raw reads from Fragaria chiloensis and Raphanus sativus or Arabidopsis thaliana leaf tissue (NCBI SRA accession SRP018034) were TPM values of pollen- and chloroplast-specific genes and enrichment ratios between genes of each group in all three RNAseq datasets and were recorded into Excel. The data from the RT-PCR experiment on Raphanus sativus pollen and leaves included raw Ct values (i.e., technical replicate Ct values) of pollen- and chloroplast-specific genes in each tissue type and were output into an .sds file by the GRC at the University of Pittsburgh, which we copied directly into Excel. We calculated the relative expression of the genes in each tissue type from the raw Ct values using the double delta method in Excel. Lastly, we report the custom forward and reverse primers used to detect the expression of the pollen- and chloroplast-specific genes, which were designed as described above in the "Data Collection" section of the "Software and Code" section of the Reporting Summary.

All authors participated in at least one type of data collection.

Timing and spatial scale

One pollen sample was collected from each plant species chosen from the California Coastal region March 4 - 8, 2018; from Central Appalachia April 20 - 24, 2018; from the California Grasslands May 5 - 17, 2018; and from the Eastern Deciduous Agro-forest

	Interface August 7 - 11, 2018. We chose to sample at these times because we wanted to include as many diverse plant species in our study as possible. We collected the pollen samples in the morning or early afternoon to avoid competition with pollinators.<br><br>The Raphanus sativus leaf tissue was collected from the California Coastal region in March 2018 at the same time the pollen was collected from that plant species.
Data exclusions	No data were excluded from this study.
Reproducibility	Although this study was not experimental, we have disclosed all software that was used (see the "Data Collection" section of the "Software and Code" section of the Reporting Summary) and all output (Supplementary Datasets 1 and 2) from the viral discovery pipeline (Pickaxe) so that reviewers and readers can easily follow how decisions were made concerning the identification of known viruses and novel viral genomes found to be in association with pollen.<br><br>In addition, any R code generated for standard data analysis for this manuscript is available upon request and the Pickaxe code is available upon request or can be accessed at https://github.com/pcantalupo/pickaxe or at Zenodo: doi: 10.5281/zenodo.5718362.
Randomization	Randomization was not relevant to this study. This study was not experimental/manipulative (i.e., there were no treatments or groups). Instead, we identified known viruses and novel viral genomes in association with pollen and related those results to plant traits and human land use within the regions.
Blinding	Blinding was not relevant to this study. This study was not experimental/manipulative (i.e., there were no treatments or groups). Instead, we identified known viruses and novel viral genomes in association with pollen and related those results to plant traits and human land use within the regions.

Did the study involve field work? ☒ Yes ☐ No

Field work, collection and transport

Field conditions	No environmental parameters were relevant to this study, but all pollen sampling was done in fair weather (i.e., days on which no precipitation was falling). We collected the pollen samples in the morning or early afternoon to avoid competition with pollinators.
Location	The four regions in the United States from which we collected pollen were the California Grasslands, the California Coast, Central Appalachia, and the Eastern Deciduous Agro-forest Interface. All sampling sites in both the Grassland and Coastal regions were in California. The sampling sites in Central Appalachia were in North Carolina and Georgia, and all those in the Eastern Deciduous Agro-forest Interface were all in Pennsylvania. The GPS coordinates for sites in the California Grasslands region were: 38.866142, -122.453171; 38.857691, -122.408093; 38.8861, -122.5102; 38.8864, -122.5084; 38.861049, -122.422534; 38.859634, -122.411384. The GPS coordinates for sites in the California Coast region were: 36.0582, -121.5904; 38.3178, -123.0703; 38.3262, -123.008; 37.5516, -122.5123; 38.3162, -123.0685; 38.3334, -122.97. The GPS coordinates for sites in Central Appalachia were: 35.6217, -81.5784; 36.1192, -81.8332; 35.9011, -81.8033; 35.6019, -81.6272; 35.7311, -81.9031; 34.9786, -83.4784. The GPS coordinates for sites in the Eastern Deciduous Agro-forest Interface were: 41.6188, -80.4441; 41.5734, -80.4974; 41.6188, -80.4441; 41.6009, -80.4568; 41.5734, -80.4974; 41.6033, -80.4563.
Access & import/export	Many of the pollen samples (and the leaf sample) in this study were collected from public roadsides. However, some from the California Grasslands were collected from the University of California McLaughlin Natural Reserve, and some from the Eastern Deciduous Agro-forest Interface were collected from the University of Pittsburgh Pymatuning Laboratory of Ecology. We had permission to sample in both places. In addition, we obtained permission from the USDA Forest Service to sample in the Till Ridge Cove area of the Chattahoochee-Oconee National Forest from April 18 to April 25, 2018, though some of the Central Appalachia pollen samples were also collected from public roadsides.<br><br>All pollen samples were preserved on dry ice in the field. They were also shipped overnight on dry ice to the University of Pittsburgh, where they were stored at -80C until the RNA extraction phase of this study. The Raphanus sativus leaf discs were immediately submerged in RNAlater (Invitrogen, ThermoFisher Scientific, Waltham, MA, USA) in the field and kept at room temperature for seven days until frozen and shipped overnight on dry ice to the University of Pittsburgh, where they were stored at -80C until the RNA extraction phase of this study.
Disturbance	No short- or long-term disturbance to any habitat was caused by this study.

Reporting for specific materials, systems and methods

We require information from authors about some types of materials, experimental systems and methods used in many studies. Here, indicate whether each material, system or method listed is relevant to your study. If you are not sure if a list item applies to your research, read the appropriate section before selecting a response.

Materials & experimental systems

n/a	Involved in the study
x	Antibodies
x	Eukaryotic cell lines
x	Palaeontology and archaeology
x	Animals and other organisms
x	Human research participants
x	Clinical data
x	Dual use research of concern

Methods

n/a	Involved in the study
x	ChIP-seq
x	Flow cytometry
x	MRI-based neuroimaging
